# Expansion of the Yeast Modular Cloning Toolkit for CRISPR-Based Applications, Genomic Integrations and Combinatorial Libraries

**DOI:** 10.1021/acssynbio.1c00408

**Published:** 2021-12-03

**Authors:** Maximilian Otto, Christos Skrekas, Michael Gossing, Johan Gustafsson, Verena Siewers, Florian David

**Affiliations:** † Department of Biology and Biological Engineering, Chalmers University of Technology, Gothenburg SE-41296, Sweden; ‡ Novo Nordisk Foundation Center for Biosustainability, Chalmers University of Technology, Gothenburg SE-41296, Sweden; § Discovery Sciences, Biopharmaceuticals R&D, AstraZeneca, Gothenburg SE-43150, Sweden; ∥ Wallenberg Center for Protein Research, Chalmers University of Technology, Gothenburg SE-41296, Sweden

**Keywords:** Saccharomyces cerevisiae, toolkit, modular cloning, gRNA array, genomic integration, library construction

## Abstract

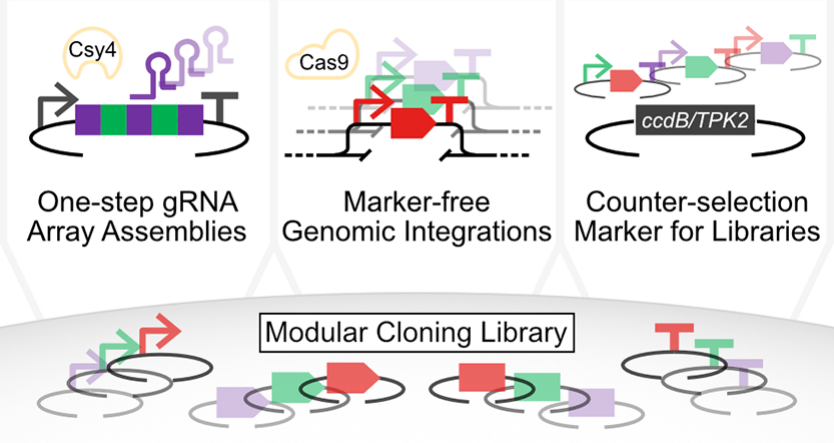

Standardisation of genetic parts has become a topic of increasing interest over the last decades. The promise of simplifying molecular cloning procedures, while at the same time making them more predictable and reproducible has led to the design of several biological standards, one of which is modular cloning (MoClo). The Yeast MoClo toolkit provides a large library of characterised genetic parts combined with a comprehensive and flexible assembly strategy. Here we aimed to (1) simplify the adoption of the standard by providing a simple design tool for including new parts in the MoClo library, (2) characterise the toolkit further by demonstrating the impact of a BglII site in promoter parts on protein expression, and (3) expand the toolkit to enable efficient construction of gRNA arrays, marker-less integration cassettes and combinatorial libraries. These additions make the toolkit more applicable for common engineering tasks and will further promote its adoption in the yeast biological engineering community.

## Introduction

The recent developments in the fields of synthetic biology and metabolic engineering have enabled us to genetically manipulate microorganisms in order to produce industrially relevant chemical compounds in a more sustainable way. This can be achieved by both, manipulating the endogenous metabolism of the cell and/or by introducing novel genes. The yeast *Saccharomyces cerevisiae* serves both as a model organism and a widely used cell factory for production of biofuels, pharmaceuticals and other industrially relevant compounds.^1−3^

While standardisation in traditional engineering disciplines, like mechanical or electrical engineering, is considered indispensable, standardisation in biology is arguably still in its infancy.^4^ Nonetheless, the emergence of synthetic biology, driven by advances in molecular cloning and genetic engineering, has put standardisation center stage. A comprehensive standard for genetic parts could bring a variety of advantages, including but not limited to more predictable and reproducible results, acceleration or automation of the engineering process, and simplifying the sharing of constructs. The overall idea is to standardise basic genetic elements such as promoters, coding sequences, terminators etc. and collect them as a library of parts. These parts can be assembled to form devices or possibly even whole synthetic biological systems in the future.^5−7^ Examples for assembly standards that have been adapted for multiple organisms, including *S. cerevisiae*, are GoldenBraid and Modular Cloning (MoClo).^8−13,74,75^ Both approaches are based on Golden Gate assembly reactions,^14,15^ using type IIS restriction enzymes, which have spatially distinct recognition and cleavage sites.^16^ The use of type IIS restriction enzymes allows for directional and scarless ligation of multiple fragments in a one-pot reaction. MoClo is a hierarchical system where each plasmid is categorised in one of three distinct levels.^8,13^ Level-0 plasmids are part plasmids; they all have the same plasmid backbone and contain a single part (e.g. a promoter) and make up the MoClo part library. Level-1 plasmids are assembled from level-0 part plasmids and, in the yeast MoClo toolkit, contain a yeast and *Escherichia coli* compatible plasmid backbone with a single yeast expression cassette.^8^ Level-1 plasmids can be assembled from level-0 plasmids due to the standardised overhangs for each type of part, for example all promoters have the same overhang. Level-2 plasmids are constructed from level-1 plasmids and contain multiple yeast expression cassettes (up to six cassettes in total). For the assembly of level-2 plasmids dedicated assembly connector parts are used.

The comprehensive yeast MoClo toolkit from Lee et al.^8^ includes 94 part plasmids containing promoters (so called type-2 parts), commonly used genes (type-3 parts), terminators (type-4 parts), yeast selection markers (type-6 parts), yeast origins of replication (ori) (type-7 parts), bacterial selection markers with ori (type-8 parts) and assembly connectors (type-1 and -5 parts). The authors demonstrated the potential of their toolkit for a variety of engineering approaches, including plasmid construction and genomic integrations.

One goal of this study is to further expand this toolkit for additional CRISPR/Cas9 applications. CRISPR/Cas9 is a genetic engineering technology that originates from an immune system present in bacteria and archaea. It enables the introduction of double-strand breaks (DSBs) in specific loci of the DNA. This is achieved by the endonuclease Cas9, which forms a complex with a guide RNA (gRNA). The first 20 bp of the gRNA (spacer) target a specific sequence in the genome through base-pairing and Cas9 is activated if the target 20 bp sequence is followed by the protospacer adjacent motif (PAM) NGG.^17,18^ The remainder of the gRNA represents the scaffold that recruits the Cas9 for DNA cleavage. This technology has been successfully implemented in *S. cerevisiae*(19) and has speeded up processes such as introduction of mutations,^19^ multiplexed gene deletion,^20,21^ chromosomal integration of genes^22,23^ and chromosomal DNA library selection.^24^ A variation of CRISPR/Cas9 has been used for sequence-specific repression (CRISPR interference or CRISPRi) or activation (CRISPR activation or CRISPRa) of gene expression. Here, a catalytically inactive mutant of Cas9 (dCas9) is fused with transcription repressors or activators and this technology has been successfully used for gene expression fine-tuning and pathway control.^25−29^ Catalytically inactive dCas9 or single strand cutting nickases (nCas9) can be also fused with nucleotide diversification enzymes such as cytidine deaminase (AID) or polymerases for targeted gene diversification.^30−33^ Simultaneous expression of multiple gRNAs can expand the CRISPR/Cas9-derived technologies even more by targeting multiple DNA loci at the same time. In *S. cerevisiae*, gRNA multiplexing has been achieved either by expressing the gRNAs under individual promoters^34^ or as a single transcript.^21,35,36^ The approach of using the Csy4 endonuclease to process long transcripts with multiple gRNAs and release of single gRNAs has been successfully used for deleting up to four genes in a single transformation^21^ and for multiplexing up to twelve gRNAs for fine-tuned promoter silencing with CRISPRi.^35^ The quickest method so far for assembling multiple gRNAs in a Csy4 array^35^ requires a first PCR with one phosphorylated primer, PCR product ligation and a second PCR that introduces 4 bp overhangs between the gRNAs sequences. In this study, we aim to develop a faster and simpler method for gRNA multiplexing that involves only one PCR round and does not insert additional sequences between the different gRNAs. We also propose a more standardized plasmid system for any CRISPR/Cas9-based approach, which enables both quick domain shuffling for different types of CRISPR applications and single-step cloning of either single or multiplexed gRNA.

The efficient homologous recombination machinery in *S. cerevisiae* makes genomic integrations comparatively straight-forward.^37^ CRISPR-Cas-technologies have further increased transformation efficiencies and enable reliable integration of multiple expression cassettes at different genomic loci in a single transformation step.^23^ The ease of engineering in *S. cerevisiae* is especially relevant for metabolic engineering and synthetic biology projects, which usually require multiple iterations of the design-build-test-learn (DBTL) cycle and which can require expression of over 20 genes.^38−40^ Compared to plasmid-based expression, advantages of genomic integrations include higher genetic stability, less cell-to-cell variability and no reliance on selection markers that can alter cell physiology.^8,41−43^

Integration cassettes contain 3′ and 5′ flanking regions homologous to the desired genomic loci and the cassette is integrated by homologous recombination at these two sites. Many toolkits have been developed to standardize and simplify the integration procedure. Mikkelsen and co-workers designed a toolkit of 11 integration vectors with carefully picked and characterized integration loci in the genome of CEN.PK113-11C.^44^ The loci were selected according to three criteria: (1) high expression levels observed in a GFP fusion localization database,^45^ (2) localization in large intergenic regions to minimize effect on neighbouring genes, (3) flanked by genes essential for wild-type growth to increase genetic stability (avoiding “loop out” of integration cassettes when using identical sequences in multiple cassettes). These criteria are meant to ensure that multiple gene cassettes can be integrated and expressed at high levels, while compromising neither cell fitness nor genetic stability. The characterized integration sites were used for the EasyClone integration vector set^41^ and the subsequent iteration of the vector set called EasyClone-MarkerFree. The EasyClone-MarkerFree toolkit allows the Cas9-mediated integration of up to three marker-less cassettes simultaneously^23^ and was recently expanded to include additional integration sites.^46^

Besides construction of episomal plasmids, the yeast MoClo toolkit designed by Lee and co-workers also enables construction of integration vectors.^8^ The authors validated the integration vectors’ efficiencies by targeting two auxotrophic markers as integration loci. Homology sequences included in the toolkit as level-0 part plasmids are restricted to those marker genes. Including more homologous sequences in the toolkit that do not rely on the presence and disruption of a marker gene would be valuable additions to the toolkit and would make it more applicable for many synthetic biology and metabolic engineering applications.

While the main purpose of the MoClo toolkit is to provide an assembly standard for expression cassettes, Lee and co-workers noted that the modularity of the toolkit makes it well suited for combinatorial experiments.^8^ Instead of constructing a single, defined plasmid, a combinatorial DNA library consists of a pool of plasmid variants. Multiple elements of the expression cassette, for example the promoter, coding region and terminator, are varied simultaneously.^47^ Combinatorial approaches can create high degrees of genetic diversity and, together with high-throughput screening or selection, enable rapid analysis of a large sequence space. However, transformation efficiency often limits the transfer of the *in vitro* genetic diversity of DNA libraries to *in vivo* systems. The quality of the library is therefore paramount and the amount of non-functional variants should be reduced as much as possible.

After transformation of the Golden Gate assembly to *E. coli* not all transformants will contain a vector with the desired insert, but cells might also take up an empty vector without any insert. To facilitate identification of recombinant vectors, colorimetric methods such as the blue/white screen have been developed,^48^ which relies on the cleavage of 5-bromo-4-chloro-3-indolyl-β-d-galactopyranoside (X-gal). The MoClo toolkit uses GFP and RFP as dropout markers that can be used for a green/red/white screen^8^ and does not require the addition of a chemical indicator to the media. For screening of large libraries (>10^6^ variants), however, this approach is less useful, as picking individual transformants becomes less feasible with increasing library size, making it particularly important to ensure that all vectors carry the desired insert. A selection strategy that prevents cells transformed with an empty plasmid from growing would be a suitable solution to this problem and would lead to improved library quality.

**Figure 1:**
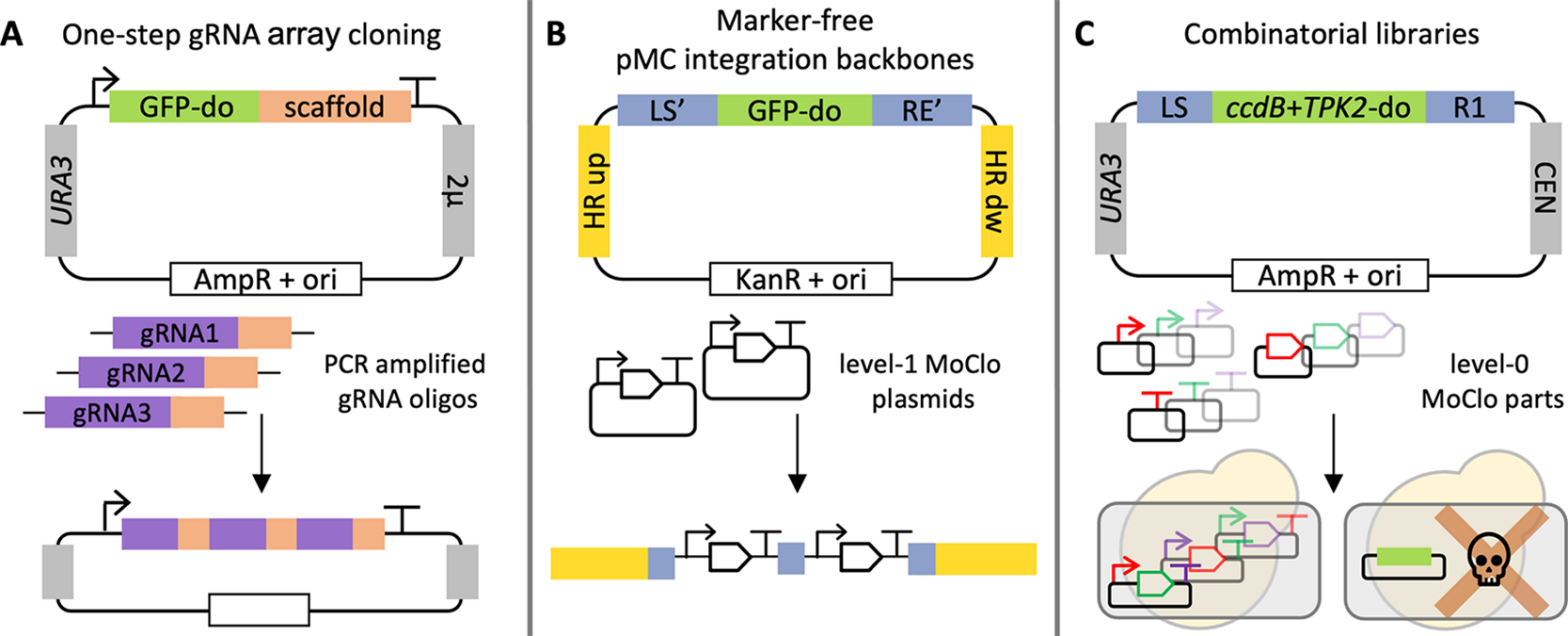
Summary of MoClo-facilitated engineering applications developed in this study. **A**: Efficient one-step assembly of gRNA arrays for CRISPR-based applications. **B**: Construction of marker-free genomic integration cassettes. **C**: Selection method to remove re-ligated, empty plasmids from combinatorial libraries. do = drop-out.

The MoClo toolkit excels at the construction of expression plasmids. Furthermore, Lee and co-workers thoroughly characterized the genetic parts of the MoClo library and their data are a valuable resource for strain design. Nonetheless, we believe that the toolkit’s full potential has not yet been reached and that it would benefit from (1) reducing the hurdle of adopting the standard by stream-lining the design of new MoClo parts, (2) further characterisation of the part library to increase predictability of strain designs, (3) enabling the toolkit to be used for common engineering tasks besides plasmid construction. Such engineering tasks include a fast assembly strategy for gRNA arrays, more possibilities for marker-less genomic integrations and a convenient method to construct combinatorial libraries (Figure 1).

## Results and Discussion

### Tool to Stream-Line the Design of New MoClo Parts

MoClo can be seen as a community-driven effort to provide standards for biological engineering. While this holds great potential for simplifying and speeding up the engineering process and furthermore improving reproducibility, the full scope of standardisation can only be achieved if said standard is widely accepted and adopted in the community.

To make it easier for researchers to adopt the yeast MoClo toolkit in their labs and to overall make the design of new genetic parts for the MoClo library more convenient, we created a design tool in the form of an Rshiny app. As input, the tool solely requires pasting the DNA sequence to be added to the library and choosing the desired prefix/suffix part type from a drop-down menu. The software will provide a list of possible forward and reverse primers with type-specific overhangs necessary to generate the new level-0 part plasmid. The user can choose the primers depending on G/C content, length and melting temperature. Furthermore, a message will be displayed if the pasted DNA sequence contains recognition sites that can interfere with MoClo (BsaI, BsmBI), BioBrick (EcoRI, XbaI, SpeI, PstI) or BglBrick (BglII, BamHI, EcoRI, XhoI) compatibility or with using the part in integration cassettes (NotI). BsaI, BsmbI and NotI recognition sites should always be removed while BioBrick and BglBrick compatibility is not always required depending on the experimental design. By being able to choose the prefix and suffix type individually, it is possible to design non-standard type parts that span over multiple part types, which for example is the case for pYTK095 which covers type-6, -7 and -8.^8^ In addition, the user can export a FASTA file that contains the level-0 part vector resulting from the Golden Gate BsmBI assembly of the PCR fragment and the entry vector pYTK001. The FASTA file can be imported into the preferred molecular biology software.

Designing primers and checking the DNA sequence for restriction sites that interfere with the Golden Gate assembly can be a tedious and error-prone process. Flawed primer design can easily lead to frame shifts in coding sequences, rendering proteins nonsensical. We believe that our design tool for new genetic parts will speed up the MoClo workflow and help individual researchers and laboratories to overcome the initial hurdle for adaptation of the standard. The tool is available here and on Zenodo (doi: 10.5281/zenodo.4944568).

### Influence of the 5′ BglII Site on Protein Production

Lee and co-workers thoroughly characterised the MoClo parts in terms of expression strength and expression pattern.^8^ Their data provide a valuable resource for designing new strains and make experiments more predictable. We aimed at expanding these data by specifically investigating the impact of the promoter 5′-non-coding region on gene expression.

MoClo expression cassettes contain a BglII site (AGATCT) in the 5′-non-coding region adjacent to the start codon, to enable BglBrick-compatibility.^49^ The composition of nucleotides −5 to −1 relative to the start codon has a significant influence on efficiency of translation initiation and consequently, the amount of protein produced. In yeast, this sequence is typically rich in adenine.^50^ In a previous study, a library of 5 × 10^5^ random 50 bp long 5′UTRs, containing all possible 1024 pentamers at positions −5 to −1, was screened in a competitive growth assay for its influence on His3 production.^51^ Interestingly, the pentamer derived from the BglII sequence (GATCT) was only ranked number 810 on the list of 1024 sequences.

**Figure 2:**
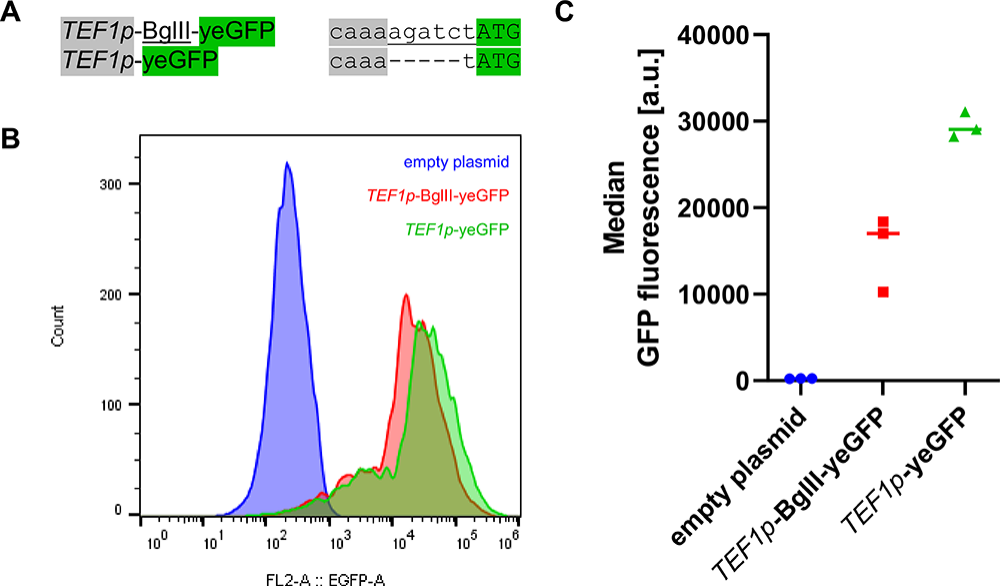
Influence of the 5′ BglII site on protein production. **A**. Sequence upstream of the start codon for *TEF1p*-BglII-yeGFP and *TEF1p-*yeGFP. Bases with grey background belong to the *TEF1p* promoter sequence, and bases with a green background belong to the yeGFP ORF. The BglII site is underlined. Note that both constructs contain a T upstream of ATG which is part of the BsaI-generated overhang for MoClo assembly (TATG). **B**. Yeast cells (CEN.PK 113-11C) containing an empty plasmid (p413TEF) plus the indicated plasmid (empty plasmid, p416TEF; *TEF1p*-BglII-yeGFP, pMG332; *TEF1p*-yeGFP, pMG333) were cultivated to log phase in synthetic minimal media, in biological triplicates. 10,000 events were recorded using a Sony SH800 cell sorter. Single cells were gated using an FSC-W vs FSC-H dot plot, and normal-sized/shaped cells were gated using a SSC-A *versus* FSC-A dot plot. Histogram of GFP fluorescence is shown for one representative replicate of each genotype. **C**. Median GFP fluorescence was determined for each replicate. Bar, median of triplicates.

To investigate the influence of the BglII site on protein production more directly, we constructed centromeric plasmids containing cassettes for expression of yEGFP^52^ under control of the strong constitutive *TEF1*p promoter, either with (pMG332) or without (pMG333) the 5′ BglII site (Figure 2). The thymine directly upstream of ATG is part of the MoClo overhang (TATG) required for Golden Gate assembly, and thus cannot be removed while maintaining yeast MoClo-compatibility. The last 4 nucleotides from the *TEF1*p promoter (CAAA), followed by thymine (T) upstream of ATG, yield pentamer CAAAT (Figure 2A), which was ranked number 92 on the list of 1024 sequences.

Cells were cultivated to log phase in a Growth Profiler, and GFP fluorescence was analyzed using a Sony SH800 Cell Sorter (Figure 2B,C). The median of fluorescence intensity was 42% lower in the presence of the BglII site. This significant reduction in protein production might make it desirable to remove the BglII site to maximize protein levels, at the cost of BglBrick compatibility, depending on the application.

### Two-Plasmid System that Facilitates Modular Cloning for CRISPR Applications

Conventional CRISPR/Cas9 applications for double strand break introduction often employ two-vector systems that express *cas9* from one plasmid and the gRNA from another.^19,23,53,54^ For other CRISPR-derived applications, it has been noted in several studies that the Cas9-derived protein (e.g. dCas9-VPR for CRISPRi) and the gRNA are all expressed from one centromeric plasmid.^28,55^ Constructing these plasmids comes with several challenges. The approach of having both the Cas9-derived protein and the gRNA encoded on the same plasmid makes the engineering of the *cas9*-derived gene (e.g. exchanging fused domains, introducing new Cas9 variants, changing promoters/terminators) more complex and time consuming due to the large size of the vector. Moreover, PCRs even with high fidelity polymerases increase the risk of mutations and also demand sequencing of the 4 kb long *cas9* gene in every new construct. Each new gRNA has to be added in every single new plasmid separately, which prolongs the engineering time even further. In addition, when it comes to the expression level of gRNAs, it has been reported that in *S. cerevisiae*, it is optimal to express the gRNAs from high-copy plasmids.^19,56−58^ On the contrary, *cas9* expression under strong promoters from a high-copy plasmid has shown to have negative effects on cell growth in some studies,^24,59^ also caused by off-target activity.^19^ These effects could also be relevant for other Cas9-based approaches that involve dCas9 or nCas9 and expression systems should be considered carefully.

**Figure 3:**
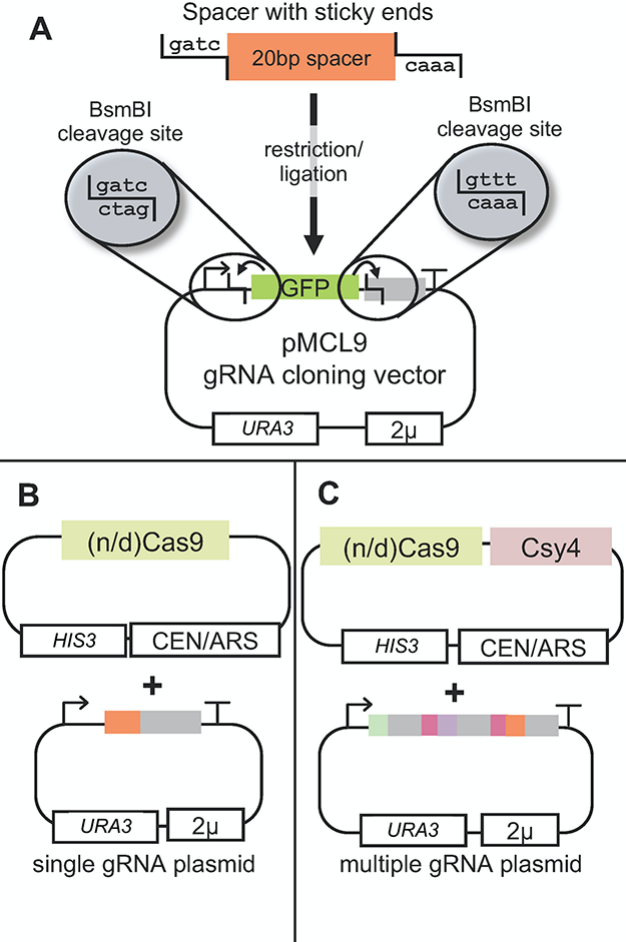
Overview of the two-plasmid system for CRISPR applications and the single-step assembly of multiplexed gRNAs. **A**. The universal gRNA cloning vector pMCL9 contains a *URA3* marker, a 2μ origin and a GFP gene flanked by two BsmBI sites. The 5′ BsmBI site is located downstream of the *SNR52* promoter, the 3′ BsmBI site is located upstream of a gRNA scaffold sequence and a 20 bp spacer with compatible sticky ends can be inserted. **B** + **C**. Design for single (**B**) or Csy4-multiplexed (**C**) gRNA expression. *Csy4* and *cas9* cassettes can be easily combined in one level-2 vector.

Here we suggest a setup where the gRNA(s) are expressed from a high-copy 2μ plasmid and the Cas9 or Cas9-derived proteins from a low-copy centromeric plasmid to ease construction and avoid toxicity and off-target effects. A modular cloning system specialized for CRISPR can facilitate the construction of such plasmids. The yeast MoClo toolkit from Lee et al.^8^ already contains a cassette for integration of a gRNA encoding sequence with BsmBI. Nonetheless, this cassette cannot be used for the construction of a single gRNA cloning vector with the MoClo toolkit because it has BsmBI sites, which are also present in the connector sequences. We constructed the new gRNA cloning plasmid pMCL9, which contains the yeast *URA3* marker, the 2μ replication origin and the ampicillin resistance gene from the MoClo kit, an RNA polymerase III promoter (*SNR52*p), a BsmBI cloning cassette with the GFP gene from the MoClo kit and an *SUP1*t-*CYC1*t terminator (Figure 3A). This plasmid can be combined with any *cas9* centromeric plasmid with another auxotrophy marker (e.g. *HIS3*, *LEU2*) or resistance marker (e.g. *kanMX, natMX*). In this study, we paired the *URA3*-based gRNA expression vector with a *HIS3*-based *cas9* expression vector (Figure 3B). Both plasmids can be constructed according to the MoClo principle, which enables the shuffling of multiple domains, which is useful especially for applications such as CRISPRi/CRISPRa and CRISPR base editing (Supporting Figure 1B). A part plasmid dCas9_3a can be used to create multi-domain cassettes by modular cloning. Then, a single domain can be fused to dCas9 as a 3b part or two domains as 3b and 4a parts. The design of parts can be facilitated by our part design tool described above. The *cas9*-derived expression cassettes can be constructed with connectors LS and R1 and then combined with an L1-Csy4-RE plasmid, thereby forming a multi-cassette plasmid with both the *cas9*-derived cassette and the *csy4* cassette (Supporting Figure 1A). This enables the use of any *cas9*-derived cassette with a Csy4-multiplexed gRNA array (Figure 3C).

In summary, we propose a new design of *cas9* vectors. A first centromeric vector will express Cas9 or any Cas9-derivative of choice and a second 2μ vector will serve as Golden Gate cloning vector of spacer gRNA sequences. This design will separate the molecular engineering of the *cas9* expression cassettes and the guide RNAs, enabling the development of more complex experimental designs, such as testing multiple Cas9-derived proteins with the same gRNAs in a shorter time and developing multiplexed gRNA arrays.

### Single-step Assembly of Multiple gRNA Arrays

**Figure 4:**
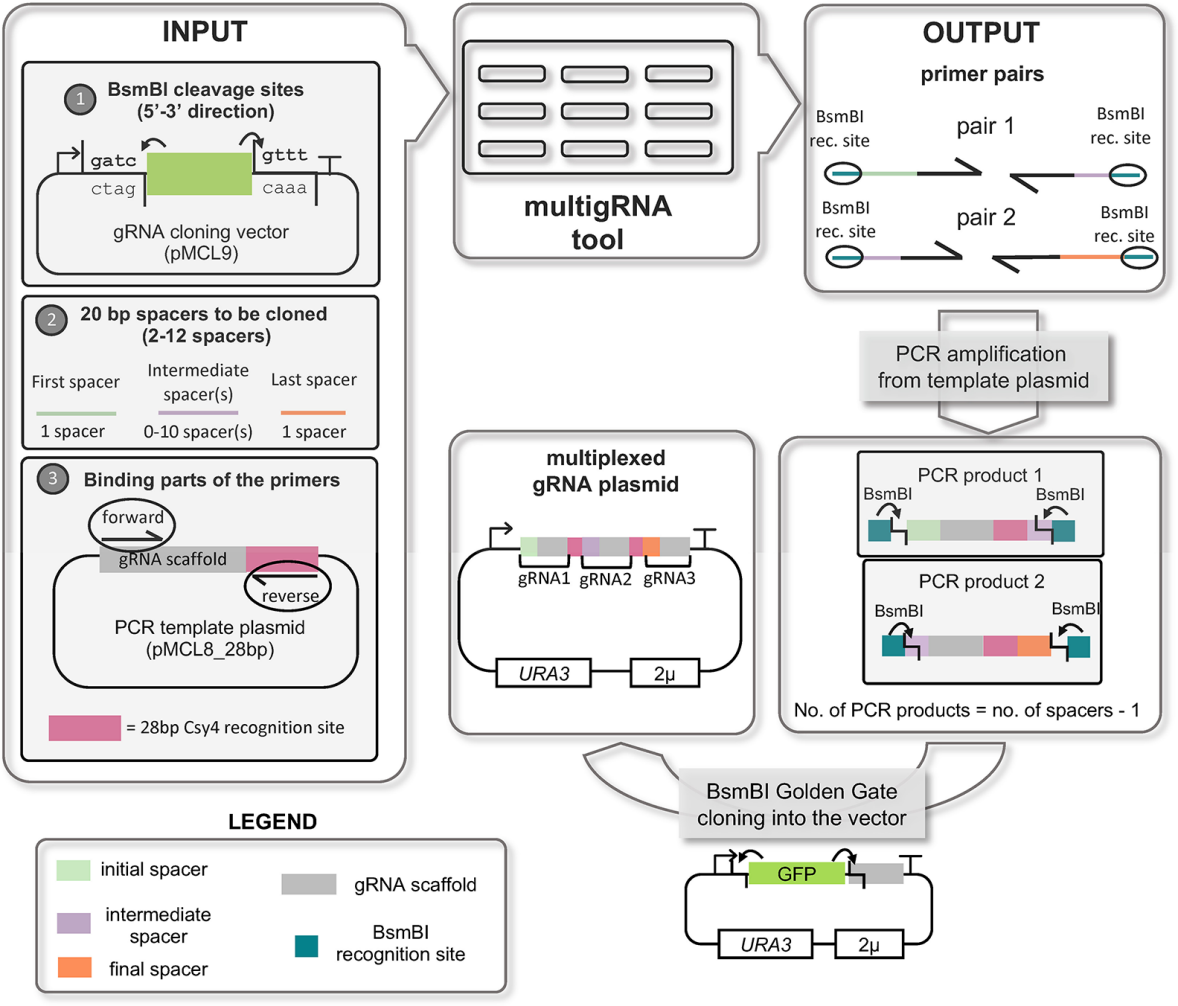
Overview of the multigRNA tool for one-step computer-aided cloning of multiple gRNA arrays. The tool is built to clone multiple gRNAs into a cloning vector that has BsmBI sites. Vector pMCL9 also contains a GFP marker between the two BsmBI sites. The cloning fragments are PCR products resulting from a template sequence that consists of a gRNA scaffold sequence followed by the *csy4* sequence that separates the gRNAs. Our template is a plasmid (pMCL8_28 bp) and the separating sequence is the 28 bp sequence that is recognised by the endonuclease Csy4. The input of the tool consists of: (1) The BsmBI cleavage sites in the cloning vector with direction 5′-3′, (2) The specific gRNA spacer sequences that are about to be multiplexed in a single transcript and (3) The binding parts of the primers. The output is primer pairs that will be used for the creation of the PCR products. The PCR products are then cloned into the vector via BsmBI Golden Gate reaction.

The gRNA cloning vector pMCL9 with BsmBI sites enables the cloning of gRNA arrays with Golden Gate cloning. Such cloning approaches have been already reported, using as cleavable RNA sequences between the different gRNAs either Csy4 recognition sequences,^35^ tRNAs^36^ or self-cleaving ribozymes.^60,61^ In this study, we expand this toolbox and introduce a single-step method for cloning gRNA arrays with Csy4 recognition sites. The cloning is performed with PCR fragments that contain outer BsmBI sites, which produce unique sticky ends in the Golden Gate reaction. The template for those PCR fragments is a plasmid containing the DNA sequence of the scaffold gRNA sequence followed by a gRNA-separating sequence of choice. In this study we used as a template the plasmid pMCL8_28 bp, which contains a scaffold gRNA sequence followed by a 28 bp Csy4 recognition sequence. We developed the online toolkit MultigRNA (available here) for computer-aided design of the primers for this gRNA multiplexing method. This toolkit has three inputs: (1) forward and reverse binding parts of the primers based on the PCR template that is used, (2) 5′ and 3′ BsmBI-created overhangs in the destination vector, with direction 5′->3′ and (3) 2 to 12 gRNA spacers of choice that need to be multiplexed in a single transcript. The MultigRNA tool automatically designs primers for this method and prevents the formation of sticky ends with 3 nucleotides in common, something that can lead to incorrect assemblies.^14^ This tool can be used to design primer sequences for cloning arrays encoding up to 12 gRNAs using the spacer sequences and the sticky ends of the cloning vector of choice as input. The first and the last spacers of the gRNA array are complete in the resulting fragments and the intermediate gRNAs (0 to 10 intermediate gRNAs can be included) are split by the tool, following the principle of having less than three common nucleotides between the resulting sticky ends. The fragments are then cloned into the vector of choice, in our case pMCL9, using Golden Gate assembly. This method requires only one round of PCRs and includes automation of primer design to ensure successful cloning of the gRNA array. The resulting plasmid can be used with any other Cas9-Csy4 expression plasmid using the two-plasmid system described in the previous section. A graphical summary of multigRNA tool can be seen also in Figure 4.

**Figure 5:**
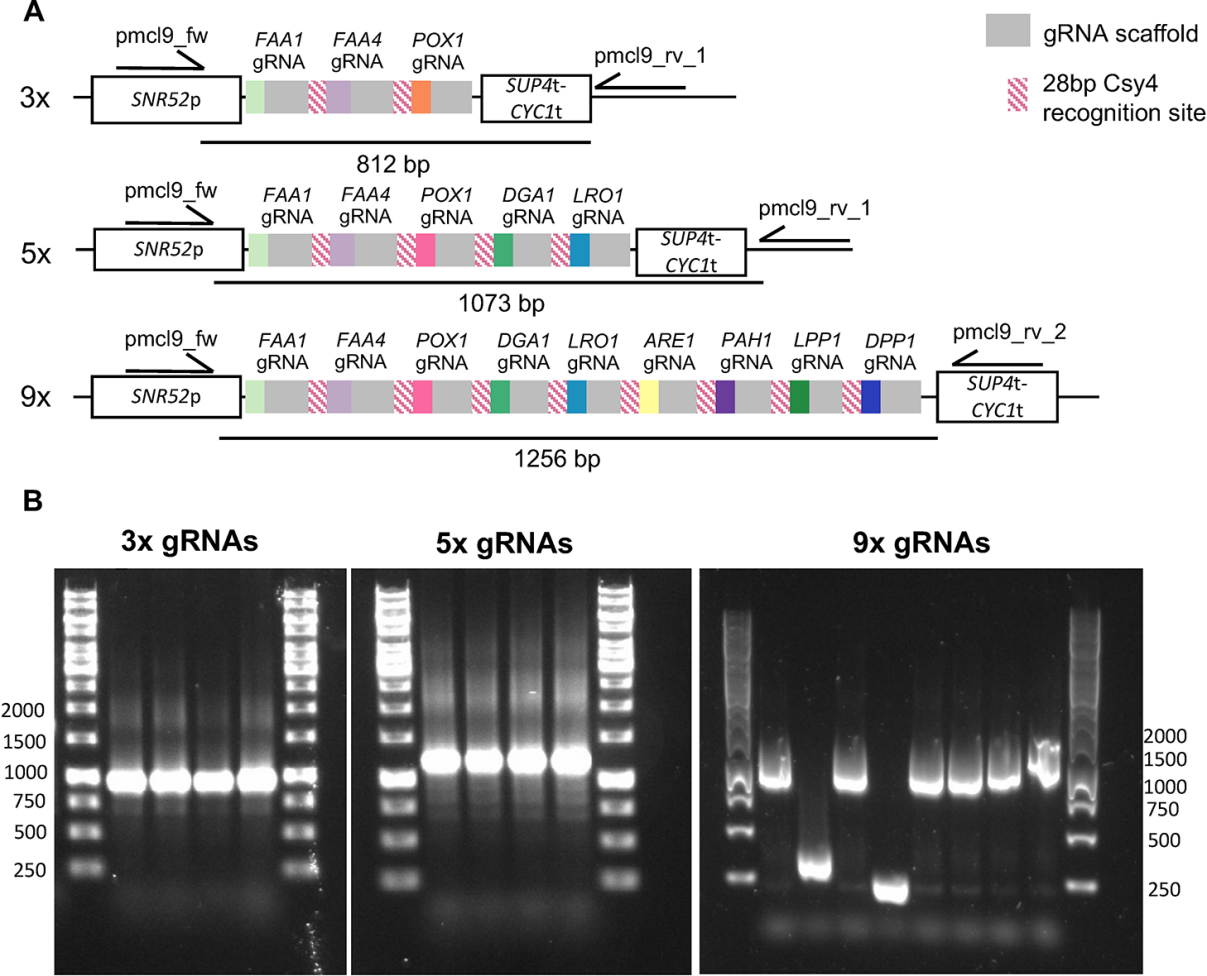
Efficiency of computer-aided gRNA multiplexing **A**. Overview of the arrays with three (3×), five (5×) and nine (9×) gRNAs multiplexed along with the primers used for colony PCR verification for each construct. Band sizes which indicate correct constructs are written under each construct. **B**. Colony PCR results for each multiplexed gRNA construct. For the 3× and 5× multiple gRNA constructs, four colonies were screened. For the 9× gRNA construct, eight colonies were screened.

Our assembly method was tested *via* one-step cloning of arrays containing three, five and nine different gRNAs. For this, we used nine gRNAs that target nine *S. cerevisiae* genes whose deletion leads to the free fatty acid overproducer yeast strain MLM1.0.^62^ Each one of those gRNAs had previously been used for the deletion of one yeast gene along with one repair fragment per gene. Using our multigRNA tool and Golden Gate assembly, we cloned strings of gRNAs that target three (3×), five (5×) and nine (9×) genes into pMCL9. Using colony PCR, it was found that for the 3× and 5× gRNA arrays the cloning success rate was 100% (4 correct out of 4 colonies tested) and for the 9× gRNA array the cloning success rate was 75% (6 correct out of 8 colonies tested) (Figure 5). Sequencing results for clones that gave a correct band in the colony PCR were correct in all cases. Thereafter, we tested the efficiency of those gRNA arrays in multiple deletions by co-transforming each gRNA array plasmid along with the corresponding repair fragments into the yeast strain pre-transformed with a Cas9-Csy4 plasmid. Eight clones per experiment were tested for gene deletions by colony PCR. A maximum number of three simultaneous gene deletions was observed. The highest efficiency was 50% triple deleted clones in the 3× gRNA array. In the same array, the deletion efficiencies were almost 90% for the first two gRNAs and 62.5% for the third gRNA. In the 5× and 9× arrays, we observed significantly lower efficiencies and also only the first three and the last gRNA showed successful gene deletions (Supporting Figure 2). This can be an indication that the Csy4 system can multiplex efficiently up to four gRNAs. It also needs to be mentioned that the more genes are to be deleted, more linear DNA repair fragments have to successfully enter the cell and more homologous recombinations are required to happen simultaneously. It is possible that more gRNAs can be functional with Csy4 multiplexing in applications such as CRISPRi, CRISPRa or targeted base editing. Successful multiplexing of 12 gRNAs using Csy4 for gene silencing *via* CRIPSRi has been previously reported.^35^

Summarizing, we developed a computer-aided strategy for a single-step Golden Gate-based cloning of single transcript gRNA arrays. We have successfully tested this method in cloning up to 9 gRNAs in a single array and we did functionality testing of those arrays *via* single transformation. We could verify high deletion efficiency for three multiplexed gRNAs and the reason for poor multiple deletion performance of more genes remains to be investigated.

### Marker-Less MoClo Integration Vectors

Lee and co-workers exemplified the construction of expression cassettes for Cas9-facilitated genomic integrations using the yeast MoClo toolkit.^8^ For this purpose, type-7 parts were designed to contain 3′ homology arms while type-8b parts contain 5′ homology arms.^8^ The resulting integration plasmid can be linearised by NotI digestion. However, there are only three homology arm pairs contained in the kit, two of which target auxotrophic markers. This severely limits the possibilities of engineering strains with stable genetic alterations. The addition of more homology arms for genomic integrations would be valuable to the toolkit and make it more applicable for many synthetic biology and metabolic engineering applications. The integration sites described by Mikkelsen and co-workers^44^ have been used in several integration toolkits, including the ECMF toolkit.^23^ The sites are situated in large intergenic regions and have been chosen carefully to allow for high gene expression, while maintaining normal cell growth and decreasing genome instability when using repeated sequences in multiple integration cassettes.^44^

We constructed level-0 part plasmids containing the individual 3′ or 5′ homology arms from the 11 integration plasmids of the ECMF toolkit.^23^ PAM sites had previously been removed from the homologous sequence to enable integrations using Cas9.^23^ For adapting the homology arms for the MoClo toolkit, in some cases BsmBI sites had to be removed from the homology sequences by introducing a point mutation (the sequences do not contain any BsaI sites). The long homology arms of 500 bp allow for high integration efficiency as well as specificity. However, reliable integration of cassettes with 10-fold shorter homology arms has been demonstrated before^63^ and the introduced point-mutations are unlikely to affect transformation efficiencies. The constructed level-0 part plasmids were named pMC7-“Y”dw and pMC8b-“Y”up for the 3′ and 5′ homology arms respectively, where “Y” is the targeted integration site (Supporting Table 1).

**Figure 6:**
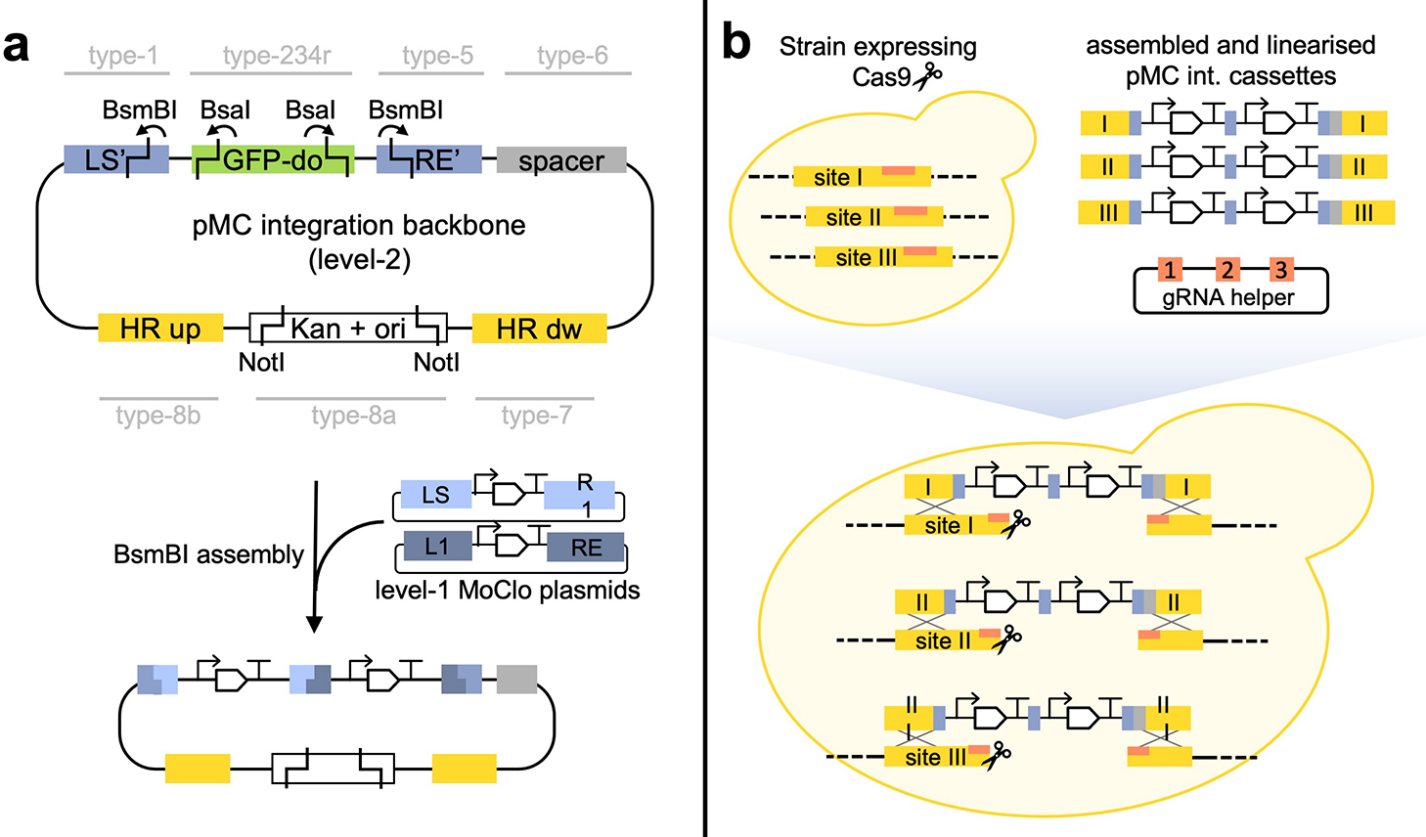
pMC integration plasmid backbones allowing for marker-free integration targeting 10 well-characterised genomic loci. **A**. Illustration of the preassembled integration backbone and an example assembly of a 2-gene integration cassette. The “HR up” and “HR dw” parts contain the homology arms from the pMC7-up and pMC8b-dw part plasmids. BsaI, BsmBI and NotI restriction sites are indicated. For BsaI and BsmBI, arrows point from the restriction enzyme binding site to the cut site. NotI is used to linearise the assembled integration cassette. The connector pairs LS/R1 and L1/RE are used in the example. However, by using all available connector pairs from the toolkit, a cassette containing up to 6 genes can be assembled. **B**. Schematic illustrating multi-loci genomic integrations using the pMC integration plasmids. The linearised plasmids are transformed together with a gRNA expressing helper plasmid into a Cas9-expressing strain, as described in the ECMF toolkit.^23^ Alternatively, the gRNA helper plasmid can also contain the *cas9* gene.

Besides the level-0 part plasmids, we also constructed level-2 integration vectors with the 234r-GFP dropout (pYTK047^8^). These pre-assembled integration vector backbones contain the LS′ and RE′ connectors, as well as a new type-6 spacer, pMC6-spacer (containing a short, innate DNA sequence), which replaces the yeast marker and enables marker-less integrations (Figure 6A). The plasmids were named pMC-“Y”, where “Y” is the targeted integration site (Supporting Table 1). A summary of the constructed pMC integration vectors including gRNA helper plasmids and verification primers can be found in Supporting Table 2. The integration backbones can be directly used to assemble multi-gene integration cassettes from level-1 MoClo plasmids. It is furthermore possible to use the integration vector backbones to construct single gene integration cassettes by adding level-0 part plasmids containing a promoter (type-2), gene (type-3) and terminator (type-4) to the assembly reaction and digesting with BsaI, thereby replacing the 234r-GFP dropout with the promoter-gene-terminator cassette. However, this is often not advisable since the resulting expression cassette cannot be used in later assemblies due to the LS′ and RE′ connectors.

While pMC7-XII4dw was assembled without difficulty, we were not able to obtain correctly assembled plasmids for pMC8b-XII4up (containing the 5′ homologous region of XII-4) and multiple gene synthesis attempts also failed. Only after switching from a high-copy to a medium-copy *E. coli* origin of replication (ori) could the plasmid be constructed. Furthermore, when using pMC8b-XII4up for the assembly of pMC-XII4, no clones with correctly assembled plasmid were found in multiple attempts. A possible explanation for the cloning difficulties encountered with the 5′ homologous region of XII-4 is that the DNA sequence has toxic effects in *E. coli*. Still, the XII-4 integration site has been used before and no problems during the cloning procedures were reported.^23,44^ As demonstrated with pMC8b-XII4up, it might be possible to assemble pMC-XII4 by switching from a high-copy to a medium-copy ori, however we did not pursue the construction of this plasmid further.

Cas9-facilitated genomic integrations require, besides the Cas9 protein itself, the expression of a gRNA for inducing the DSB at the desired locus in the genome (Figure 6B). The ECMF toolkit utilises a two-plasmid system with one centromeric plasmid, pCfB2312, carrying the *cas9* gene and a multi-copy gRNA helper plasmid containing one or three gRNAs.^23^ After the integration of one or three integration cassettes simultaneously, the gRNA helper plasmid can be removed and be replaced by another gRNA helper plasmid during the next transformation step. In our laboratory, we use the gRNA helper vectors of the ECMF toolkit (available at: https://www.addgene.org/kits/borodina-easyclone-markerfree/) in combination with the integration cassettes constructed from MoClo parts. Nonetheless, the ECMF vectors are not required to utilise the pMC integration vectors described here. The *cas9* gene is readily available as level-0 part plasmid (pYTK036^8^) and plasmids carrying custom gRNA arrays can be assembled using the workflow described in this paper. This gives the user more flexibility regarding the choice of selection markers for the gRNA or Cas9 plasmid and furthermore which integration sites can be targeted for multi-cassette integrations. It is noteworthy that integrations targeting the same chromosome at multiple loci had fewer positive clones than when loci on different chromosomes were targeted.^23^ Using the pMC8b and pMC7 part plasmids, custom integration plasmids can be assembled, for example if an integration cassette containing a selection marker is required or other MoClo assembly connectors beside LS′ and RE′ are desired.

As mentioned before, the homology arms used here are well characterised^23,41,44^ and have been used in many metabolic engineering studies (see citing literature of Jessop-Fabre et al.^23^ for examples). Several of the pre-assembled pMC integration plasmids have already been successfully used in our laboratory (data not shown) and we further confirmed their suitability by comparing a triple integration using the ECMF plasmids to a triple integration using the MoClo plasmids targeting the same integration sites. Very similar results were achieved in this direct comparison (Supporting Table 3).

The ECMF integration plasmids contain a USER cloning site.^23,64^ In this cloning technique, the same primer overhangs can be used for different promoters and genes, respectively, which provides some form of standardisation and previously used genetic parts can be re-used to construct new plasmids. However, this is not comparable with the comprehensive MoClo assembly standard with dedicated part types for each plasmid element and the extensive part library constructed by Lee et al.^8^ which was further expanded by this and other studies. When using all currently available assembly connector pairs, cassettes with up to 6 expression cassettes can be constructed in theory.

In summary, we constructed level-0 MoClo part plasmids for the 3′ and 5′ homology arms of all 11 integration sites described by Mikkelsen et al.^44^ and furthermore constructed level-2 GFP-drop out vectors for 10 of the integration sites, which streamline multi-gene, multi-loci, marker-less genomic integrations using CRISPR-Cas9.

### Vectors for Construction of Combinatorial DNA Libraries in Yeast

Studies show that combinatorial DNA libraries are a valuable tool to tackle and explore the large solution spaces encountered in biological engineering^47,65^ and due to its modularity the MoClo approach is uniquely suited for constructing such libraries.^8^ The green/red/white screening of the MoClo toolkit is useful to differentiate correctly assembled plasmids from re-ligated, empty plasmid backbones, but it is not suitable for screening a combinatorial library with thousands or millions of variants.

To overcome this limitation, the GFP dropout cassette can be exchanged for a cassette containing a toxic gene dropout, thereby allowing for growth-based selection instead of colorimetric screening. Transformants with vectors that still contain the toxic gene will not be able to proliferate, resulting in removal from the pool of transformants. One well-characterized example is the bacterial gyrase inhibitor CcdB, which has been used to facilitate cloning by eliminating transformants containing non-recombinant vectors.^66^ This system requires CcdB-resistant bacterial strains for initial construction of the toxic dropout vectors.

The bacterial CcdB toxin is not functional in yeast. To facilitate construction of combinatorial libraries directly in *S. cerevisiae*, we evaluated *TPK2* as a potential toxic marker. *TPK2* encodes the catalytic subunit of cAMP-dependent protein kinase. Overexpression of *TPK2* has been identified as toxic in a high-throughput screen.^67^ We constructed vectors encoding *ccdB* and *TPK2*, with expression of the latter driven by either the constitutive *TEF1*p promoter (pMG343) or the galactose-inducible *GAL1*p promoter (pMG344). First, functionality of the CcdB toxin in bacteria was confirmed (Table 1A). Transformation of the vectors encoding CcdB into CcdB-sensitive *E. coli* cells resulted in no transformants, while transformation of the empty control vector (p416TEF) was successful. Transformation into CcdB-resistant cells was successful for all vectors. Next, we evaluated the toxicity of *TPK2* overexpression in yeast (Table 1B)*.* Transformation of vectors containing *TPK2* resulted in no transformants on SG-Ura plates. Colonies were obtained for *GAL1*p-*TPK2* on SD-Ura plates, where expression of *TPK2* under *GAL1*p is repressed due to the presence of glucose. Taken together, these data demonstrate that a dual *ccdB* + *TPK2* toxic dropout is functional in bacteria and yeast, with *ccdB* causing toxicity in bacteria, and *TPK2* causing toxicity in yeast. This dual-purpose toxic dropout adds flexibility during library construction, making it possible to change hosts without requiring changes to DNA parts or cloning strategy. We demonstrated functionality of the toxic dropouts for level-1 plasmids, enabling combinatorial libraries for a single gene of interest. This strategy can easily be adapted for multi-step pathway optimization by using level-2 plasmids with the *ccdB* + *TPK2* toxic dropout.

**Table 1:** Toxicity of CcdB and Tpk2 **A.** 10 ng of Plasmid^a^ were Transformed into either *ccdB* sensitive^b^ or *ccdB* Resistant^c^*E. coli*. **B**. 100 ng of Plasmid^a^ were Transformed into Yeast^d^ and Plated on Either SD-Ura (Glc) or SG-Ura (Gal) Plates

A			
cfu/μg DNA	empty	*TEF1p*-*TPK2*+*ccdB*	*GAL1p*-*TPK2*+*ccdB*
*ccdB* sensitive	0.41 ± 0.03×10^7^	0	0
*ccdB* resistant	1.07 ± 0.32×10^7^	1.88 ± 0.37×10^7^	1.70 ± 0.19×10^7^

B			
cfu/μg DNA	empty	*TEF1p*-*TPK2*+*ccdB*	*GAL1p*-*TPK2*+*ccdB*
Glc	0.93 ± 0.32×10^4^	0	1.08 ± 0.18×10^4^
Gal	1.38 ± 0.03×10^4^	0	0

a empty, p416TEF; *TEF1p*-*TPK2*+*ccdB*, pMG343; *GAL1p*-*TPK2*+*ccdB*, pMG344.

b NEB 5-alpha competent *E. coli* (high efficiency).

c Invitrogen One Shot *ccd*B Survival 2 T1^R^ competent cells.

d CEN.PK 113–11C.

## Conclusion

The Design-Build-Test-Learn cycle followed in most biological engineering projects is inherently time-consuming and laborious. The adaptation of standards in biological engineering holds great potential to decrease the time spent on the Build-phase of the cycle by making parts easier to share and directly useable in a drop-in manner. However, by providing more predictable and reproducible results the overall number of cycle iterations required could be greatly reduced, which would have an even greater impact on productivity thereby reducing costs.

In the present study we improved upon the existing yeast MoClo toolkit^8^ by (1) simplifying the design of new MoClo parts *via* an Rshiny app, (2) investigating the negative impact of a BglII site on gene translation and (3) expanding the engineering capabilities of the toolkit. Said engineering capabilities include a protocol for efficient construction of Csy4 gRNA arrays, more possibilities for genomic integrations and a selection strategy for removing non-recombinant plasmids from combinatorial libraries. Having a standardised framework for these common engineering tasks results in a synergistic effect where parts can be re-used and adapted for different experimental designs, instead of just being re-used for constructing other expression cassettes. The engineering techniques discussed in this study furthermore complement each other. For example, it is possible to construct a combinatorial library of integration cassettes, which are then simultaneously integrated in multiple sites in the genome using a customised gRNA array plasmid.

Compared to academic laboratories, standardisation of genetic parts coupled with experimental workflows will arguably bring even greater benefits for biofoundries in which a high degree of standardisation is critical for automation of large-scale strain engineering. Workflows as the ones discussed in this study can for example facilitate the automated construction of large variant libraries and enable biofoundries to go beyond the construction of individual strains.

In summary, we believe that this study represents a step towards more standardisation in the yeast biological engineering community and make the MoClo standard more applicable and accessible.

## Materials and Methods

### Availability of MoClo Plasmids Constructed

All newly constructed MoClo plasmids are summarised in Supporting Table 1. Most plasmids are available on Add gene (depositing lab Florian David) with other plasmids available on personal request. Plasmid maps can be downloaded *via* the same link.

### Strains and Media

The yeast strain used in this study is CEN.PK113-11C (*MAT*a *MAL2-8C SUC2 ura3-52 his3*Δ). When no selection is needed yeast cells were grown in YPD medium containing 20 g·L^-1^ glucose, 10 g·L^-1^ yeast extract and 20 g·L^-1^ peptone from meat. Plasmid-carrying strains that needed selection in the absence of uracil and histidine were grown in SD-His-Ura medium containing 6.9 g·L^-1^ yeast nitrogen base without amino acids (Formedium), 0.77 g·L^-1^ complete supplement mixture without histidine and uracil (Formedium) and 20 g·L^-1^ glucose. Plasmid-carrying strains requiring antibiotics selection were cultivated in YPD containing G418 (200 mg/L, Gibco) or YPD containing G418 (200 mg/L) and Nourseothricin (100 mg/L, Jena Bioscience). For agar plates, 20 g·L^-1^ agar was added in the medium before autoclaving.

Liquid yeast cultures were grown in shake flasks in 30 °C with 200 rpm shaking. Yeast agar plates were grown in 30 °C for 2–3 days. For cloning and amplification of plasmids containing the *ccdB* cassette, *E. coli* One Shot ccdB Survival 2 T1R Competent Cells (Invitrogen) were used. All other plasmids were cloned and amplified in *E. coli* DH5α. Medium for *E. coli* was LB containing 10 g·L^-1^ sodium chloride, 5 g·L^-1^ yeast extract, 10 g·L^-1^ peptone from casein and 16 g·L^-1^ agar when preparing plates. *E. coli* liquid cultures were grown in 37 °C and 200 rpm shaking for 16–20 h and agar plates in 37 °C for 16–20 h. For plasmid selection antibiotics were added in LB in the following concentrations: ampicillin 100 mg·L^-1^, kanamycin 50 mg·L^-1^ and chloramphenicol 25 mg·L^-1^.

### Molecular Biology Reagents

T4 DNA ligase and all restriction enzymes were purchased from Thermo Fisher Scientific. Colony PCRs were performed with DreamTaq DNA polymerase (Thermo Fisher Scientific, catalogue number: EP0705). For *E. coli* colony PCR, one colony was resuspended in 50 μL dH_2_O and 1 μL was used as a template. *S. cerevisiae* DNA extraction for colony PCR was performed as described previously.^68^ For PCR amplification of the homology arms, PrimeStar Polymerase was used (Takara Bio, catalogue number: R010B). All other PCRs were performed with Phusion High-Fidelity DNA polymerase (Thermo Fisher Scientific, catalogue number: M0530). PCR protocols were followed according to the manufacturers’ recommendations. Primer sequences can be found in Supporting Table 4. PCR purifications, gel extractions and plasmid minipreps were performed using GeneJET kits (Thermo Fisher Scientific, catalogue numbers: K0702, K0692, K0502). If a plasmid was used as PCR template, 1 μL DpnI was added after the PCR and the mix was incubated for 1 h. *E. coli* transformation was performed according to the protocol described by Hanahan.^69^ For Gibson assemblies, Gibson Assembly Master Mix was used (NEB, catalogue number: E2611).

### MoClo Assembly Reactions

For Golden Gate assemblies, T4 DNA ligase (Thermo Fisher Scientific, catalogue number: EL0011) and either Eco31I (BsaI) (Thermo Fisher Scientific, catalogue number: ER0291/FD0293) or Esp3I (BsmBI) (Thermo Fisher Scientific, catalogue number: ER0451/FD0454) were used. Eco31I (BsaI) was used for assemblies of level-1 plasmids and GFP drop-out plasmids. Esp3I (BsmBI) was used for assemblies of level-0 part plasmids and level-2 multi-gene cassette plasmids. For most assemblies, the FastDigest (FD) versions of the restriction enzymes were used. To avoid misunderstandings, we refer to the restriction enzymes Eco31I and Esp3I as BsaI and BsmBI respectively in the text, as this is the nomenclature used in the original yeast MoClo paper.^8^

For the assembly reactions, approximately 20 fmol of each plasmid were added to the reaction mix containing 1 μL T4 ligase buffer, 0.5 μL T4 DNA ligase, and 0.5 μL of either BsaI or BsmBI. The reaction mixture was then topped up with Milli-Q water to a total volume of 10 μL. The assemblies were performed in a PCR cycler. Supporting Table 5 summarises the used Golden Gate assembly protocols, which were adapted from Engler and co-workers.^14^

2 μL of Golden Gate assembly reaction were added to 20 μL chemically competent *E. coli* DH5α. The cells were heat shocked for 45–60 s at 42 °C, 1 mL LB medium was added to the tube and the cells were incubated at 37 °C for 45–60 min 100–200 μL of the recovered cells were then plated on LB plates containing the relevant antibiotic and incubated overnight.

The isolated plasmids were analysed by restriction digestion for verification before being sequenced.

### Construction of CRISPR Plasmids

The gRNA cloning vector pMCL9 was constructed by ligation of six DNA fragments: (1) 1072 bp fragment from BsaI digestion of plasmid pYTK074 (*URA3*), (2) 1005 bp fragment from BsaI digestion of plasmid pYTK082 (2 micron origin), (3) 1870 bp fragment from BsaI digestion of plasmid pYTK083 (*AmpR*-ColE1), (4) BsaI digested PCR fragment amplified from plasmid pMEL10 with primers MCL27/MCL28 (pSNR52), (5) BsaI digested PCR fragment amplified from plasmid pYTK50 with primers MCL30/MCL32 (GFP cassette), (6) BsaI digested PCR fragment amplified from plasmid pMEL10 with primers MCL33/MCL34 (SUP4t-CYC1t). All the fragments were gel extracted and ligated with T4 ligase for 1 h and transformed to *E. coli*. Correct clones were identified by miniprep and double digestion with XbaI/NdeI. All pYTK plasmids were obtained from the yeast MoClo kit^8^ and pMEL10 from EUROSCARF (Frankfurt, Germany). Plasmid pMCL8 was constructed by Gibson assembly of the following PCR fragments amplified with primers: (1) MCL13/MCL14 from pYTK50 (2) MCL17/18 from pYTK50 (3) MCL15/MCL16 from pERA-109^28^ and (4) MCL19/MCL20 from pERA-109. Plasmid pMCL8_28 bp was constructed by PCR amplifying plasmid pMCL8 with the primers Csy4_fw/Csy4_rv, digesting the template with DpnI for 3 h and transformation into *E. coli*. The plasmid was verified by sequencing with primer MCL23. Plasmid LS-Cas9-R1 was constructed by combining in a BsaI Golden Gate reaction the plasmids pYTK002, pYTK013, pYTK036, pYTK053, pYTK076, pYTK081 and pYTK084. Plasmid Csy4_2-4 was constructed by combining in a BsmBI Golden Gate reaction the plasmid pYTK001 with the purified PCR product amplified from plasmid p413_Csy4NLS^21^ with primers MCL11/MCL12. L1-Csy4-RE plasmid was constructed by combining in a BsaI Golden Gate assembly the plasmids pYTK003, Csy4_2-4, pYTK072, pYTK76, pYTK81 and pYTK84. LS′-GFP-RE′ is a *HIS3* centromeric vector for cloning level-2 cassettes and it was made by combining in a BsaI Golden Gate assembly the plasmids pYTK008, pYTK047, pYTK073, pYTK076, pYTK081 and pYTK083. The plasmid Cas9_Csy4 was made by combining in a BsmBI Golden Gate assay the plasmids LS-Cas9-R1, L1-Csy4-RE and LS′-GFP-RE′. dCas9_3a plasmid was synthesized by Twist Bioscience.

### Multiplexed Gene Deletion

Csy4-multiplexed gRNA arrays were cloned into the pMCL9 vector. Cas9 and Csy4 were expressed from the plasmid Cas9_Csy4. Yeast strain CEN.PK113-11C with pre-transformed Cas9_Csy4 was transformed with 0.5 μg of the multiplexed gRNA plasmid along with 1.5 μg of each repair fragment. The repair fragments were 120 bp long and they consisted of the last 60 bp of the promoter and the first 60 bp of the terminator sequence of each gene. The diagnostic primers for verifying the gene deletions were designed to be located outside the targeting ORFs as it was described previously.^70^ The gRNAs, repair fragments and diagnostic primers had been used in a previous study^62^ and are listed in Supporting Table 6. The gRNAs were multiplexed and cloned by using the multigRNA tool and the primers used are listed in Supporting Table 4. The gene deletion verification was done by colony PCR with the primer pairs pmcl9_fw/pmcl9_rv_1 or pmcl9_fw/pmcl9_rv_2. We constructed three gRNA plasmids named as follows (in parenthesis are the genes that the gRNAs of each plasmid are targeting): 3×_gRNA (*FAA1, FAA4, POX1*), 5×_gRNA (*FAA1, FAA4, POX1, DGA1, LRO1*) and 9×_gRNA (*FAA1, FAA4, POX1, DGA1, LRO1, ARE1, PAH1, LPP1, DPP1*).

### Yeast Transformations

Yeast transformation was performed with the LiAc/PEG method.^71^ For genomic integrations, the workflow from the ECMF paper^23^ was followed. We refer to the Supporting Information of the ECMF paper for a step-by-step guide in which the procedure for genomic integrations is well summarised.

### Construction of Level-0 MoClo Vectors Containing Homology Arms

The ECMF integrations vectors^23^ were used as templates to amplify the homologous regions of the integration sites. Before construction of the level-0 MoClo vectors, BsmBI recognition sites (there are no BsaI sites in the homologous regions) were removed from pCfB2988 (X2), pCfB3034 (X3), pCfB3036 (XI1) and pCfB3038 (XII1) by introducing point mutations *via* PCR primers. The primer pairs 343/344, 345/346, 347/348, 349/350 and 351/352 were used for this purpose. The primer pairs 288/289 and 290/291 were designed to bind shortly before and after the 5′ and 3′ homologous regions respectively; this way the same primers could be used for all ECMF integration vectors. In the yeast MoClo toolkit, type 8b and type 7 plasmids can contain homologous sequences for integration cassettes.^8^ The primer pair 288/289 was designed to contain type-8b specific overhangs and the primer pair 290/291 was designed to contain type-7 specific overhangs (overhangs contain BsmBI and BsaI sites for the Golden Gate assemblies, see Supporting Information of Lee et al.^8^ for details).

The resulting PCR products were then used in a BsmbI Golden Gate assembly with the entry vector pYTK001. The type-8b plasmids containing the 5′ homologous regions were named pMC8b-“Y”up while the type-7 plasmids containing the 3′ homologous regions were named pMC7-“Y”dw, where “Y” refers to the integration site. Multiple attempts to construct pMC7-X3dw, pMC7-XII1dw and pMC8b-XII4up failed. For pMC7-X3dw and pMC7-XII1dw insertions occurred in the homologous sequence, while for pMC8b-XII4up no correctly assembled plasmids could be obtained. We therefore ordered the plasmids to be synthesized by Twist Bioscience with high-copy vector backbones containing a chloramphenicol resistance marker cassette (which is used for all level-0 MoClo plasmids). pMC7-X3dw and pMC7-XII1dw were synthetised without problems, but multiple synthesis attempts for pMC8b-XII4up failed and only after switching to a medium-copy vector backbone with chloramphenicol marker correct clones were obtained.

### Construction of Preassembled MoClo Integration Backbones with GFP Drop-Out

A new type-6 level-0 part plasmid, pMC6-spacer, was constructed containing a 37 bp non-coding DNA sequence (sequence from the spacer plasmid pYTK048). The DNA sequence was ordered as forward and reverse oligos (O1 and O2), which contain the BsmBI sites required for level-0 part assemblies and the BsaI site with type-6 specific overhangs. The annealed oligos were used in a BsmbI Golden Gate assembly with the entry vector pYTK001. Type-6 parts are normally dedicated to yeast markers and the constructed spacer can be used to assemble marker-less integration plasmids.

For the preassembled MoClo integration vectors the level-0 part plasmids pYTK008 (LS′ connector), pYTK047 (GFP drop-out), pYTK073 (RE′ connector), pYTK090 (KanR-ColE1), pMC6-spacer, pMC7-“Y”dw and pMC8b-“Y”up (where “Y” is the integration site) were combined in a BsaI Golden Gate assembly reaction (protocol ending on ligation). The resulting integration vectors are level-2 MoClo backbones with GFP drop-out that can be used to conveniently assemble multi-gene marker-less integration cassettes. The plasmids are named pMC-“Y” (where “Y” is the targeted integration site). A summary of the constructed integration vectors, their gRNA helper plasmid and their verification primer pairs can be found in Supporting Table 2. As we were not able to assemble pMC-XII4, the integration site was excluded from this toolkit.

The ECMF toolkit also includes verification primer pairs for each integration site.^23^ The 3′ primer pair from the ECMF toolkit, using the universal 3′ primer 2220, is compatible with MoClo integration vectors. However, the universal 5′ primer 2221 from the ECMF toolkit does not bind in the MoClo integration vector and a new universal 5′ primer, 287, was designed for this purpose, binding shortly downstream of the 5′ homologous region (Supporting Tables 2 and 4).

### Construction of *ccdB*/*TPK2* Toxic Drop-Out Cassette

The *TPK2* gene was synthesized without BsaI and BsmBI sites and cloned into a MoClo compatible vector by Twist Bioscience. The resulting level-0/type-3 plasmid was named pMC3-TPK2. The *ccdB* expression cassette, including the native *ccd* promoter, was PCR amplified from pXII2-ccdB (kindly provided by Uffe Mortensen, see Supporting Note 1 for amplified DNA sequence) using the primers 374/375, which include type-5 specific overhangs. Before this, a BsaI recognition site was removed from the gene by introducing a point-mutation using the primers 376/377. The PCR product of the *ccdB* cassette was used in a Golden Gate assembly reaction with pMC3-TPK2, pYTK056 (*TDH1*t) and either pYTK013 (*TEF1*p) or pYTK030 (*GAL1*p). These Golden Gate assemblies were then directly used as PCR template to construct the *ccdB*/*TPK2* drop-out cassettes either containing *TEF1*p-*TPK2* (primers 368/378) or *GAL1*p-*TPK2* (primers 372/378). The type-234r drop-out overhangs, introduced by the primers, have BsaI sites in reverse orientation (as in the GFP drop-out cassette pYTK047), so that the recognition sites remain in the correctly assembled vector. The PCR products were subsequently used in a BsmBI Golden Gate assembly with pYTK001, resulting in pMC234r-ccdB/TPK2 and pMC234r-ccdB/TPK2-GAL. The resulting level-0 vectors were used in a BsaI Golden Gate-assembly reaction with pMC-Ura-Cen-lvl1, resulting in pMG343 and pMG344, respectively. For cloning and amplification of *ccdB* containing plasmids, CcdB-resistant *E. coli* strains have to be used. We used One Shot ccdB Survival 2 T1R Competent Cells.

### Flow Cytometry

For flow cytometry, strains of *S. cerevisiae* were cultured in synthetic minimal medium with the following composition: 20 g/L glucose, 7.5 g/L (NH_4_)_2_SO_4_, 14.4 g/L KH_2_PO_4_, 0.5 g/L MgSO_4_·7H_2_O, 1 mL/L vitamin mix, 2 mL/L trace metal solution, pH adjusted to 6.5. Trace metal solution contained 15.0 g/L EDTA (disodium salt), 4.5 g/L ZnSO_4_·7H_2_O, 0.84 g/L MnCl_2_·2H_2_O, 0.3 g/L CoCl_2_·6H_2_O, 0.3 g/L CuSO_4_·5H_2_O, 0.4 g/L Na_2_MoO_4_·2H_2_O, 4.5 g/L CaCl_2_·2H_2_O, 3 g/L FeSO_4_·7H_2_O, 1 g/L H_3_BO_3_, and 0.1 g/L KI. Vitamin solution contained 0.05 g/L biotin, 0.2 g/L 4-aminobenzoic acid, 1 g/L nicotinic acid, 1 g/L calcium pantothenate, 1 g/L pyridoxine-HCl, 1 g/L thiamine-HCl, and 25 g/L myo-inositol. Pre-cultures were cultivated in synthetic minimal medium overnight. Cells were diluted in fresh media to a starting OD_600_ of 0.1 in a 96-well plate, and cultivated for 6 h in a Growth Profiler. Cells were then harvested by centrifugation, and resuspended in water. Resuspended cells were passed through a 35 μm mesh, and analysed on a Sony SH800 Cell Sorter using a 70 μm sorting chip. For each sample, 10,000 events were recorded. Doublets were identified by plotting FSC-W *vs* FSC-H and removed by appropriate gating. Abnormally large and/or granular cells were identified by plotting SSC-A *vs* FSC-A and removed by appropriate gating. After both gating steps, 85–94% of counted events were included in the analysis.

### Online Tools

The Yeast Toolkit Primer Design Tool and MultigRNA tool were both implemented as R-shiny apps.^72^ The melting temperature in the Yeast Toolkit Primer Design Tool is calculated according to the Wallace formula.^73^ The multigRNA tool is based on a heuristic greedy recursive algorithm that strives to split the gRNA sequences as close to the middle of the sequences as possible while selecting non-conflicting sticky ends. Sticky end conflicts are defined as at least three matching nucleotides, comparing both forward and reverse complement sequences. The worst-case execution time for the algorithm grows exponentially with the number of gRNA sequences, but in all practical cases that we have tested the execution time was within a few seconds. The code is available in GitHub (https://github.com/SysBioChalmers/YTKPrimerDesign) and a snapshot of the code is available in Zenodo (doi: 10.5281/zenodo.4944568).

## Supporting Information Available

Supporting Figure 1: overview of MoClo for CRISPR applications, supporting Figure 2: overview of the gene deletion approach using Csy4-multiplexed gRNA arrays, supporting Tables 1: plasmids constructed in this study, supporting Table 2: summary of constructed MoClo integration plasmids, supporting Table 3: comparison of ECMF integration vectors and MoClo integration vectors, supporting Tables 4: primers used in this study, supporting Table 5: Golden Gate protocols used in this study, supporting Tables 6: oligos and gRNA spacers used in this study, supporting note 1: sequence of *ccdB* cassette (PDF)
