# Supplementary material for: Expansion of the Yeast Modular Cloning Toolkit for CRISPR-Based Applications, Genomic Integrations and Combinatorial Libraries
Source: ACS Synth Biol. 2021 Dec 3;10(12):3461–74. doi: 10.1021/acssynbio.1c00408 (PMC8689691; doi:10.1021/acssynbio.1c00408)
Supplement: Supplementary file 1 — sb1c00408_si_001.pdf [file sb1c00408_si_001.pdf]

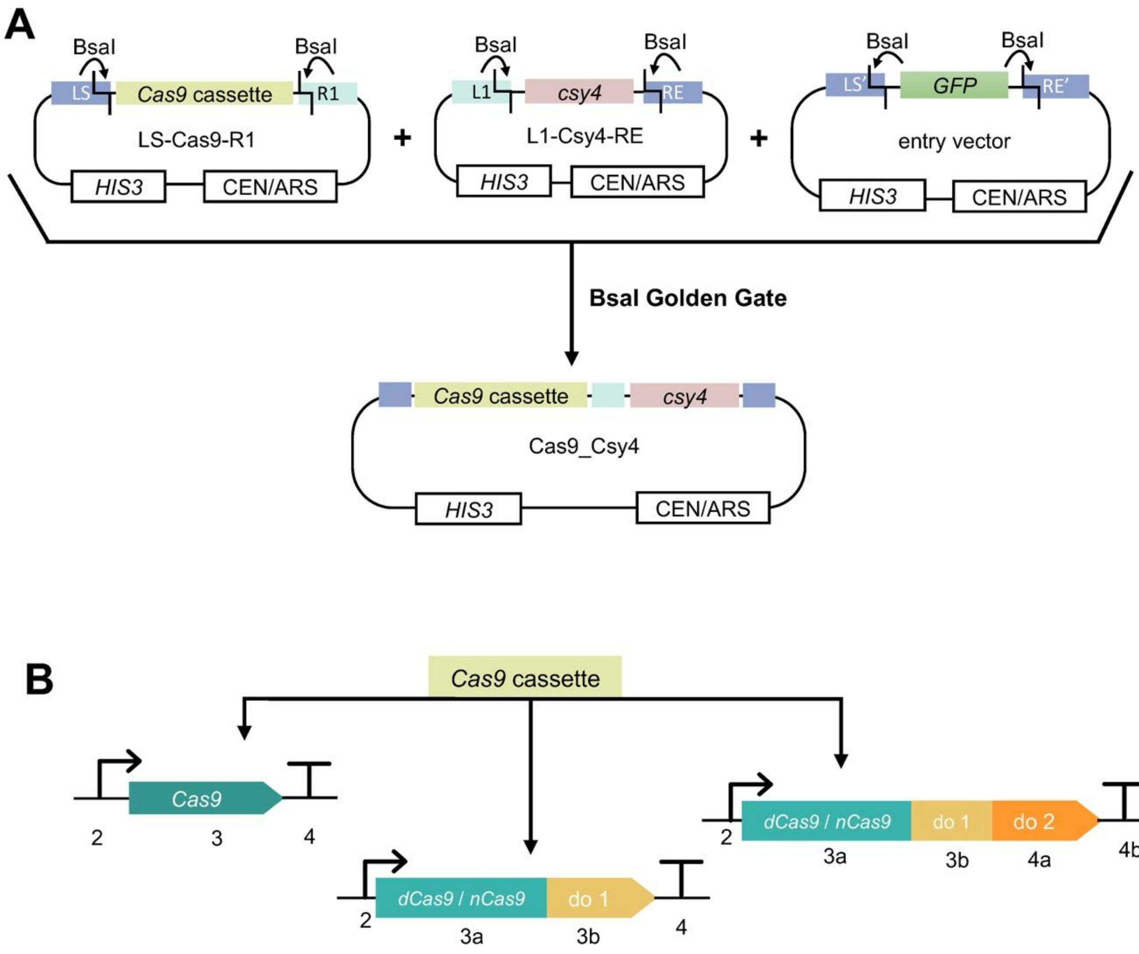

Supplementary Figure 1. Detailed overview of modular cloning for CRISPR applications **A.** Overview of the construction of Cas9_Csy4 level-2 plasmid. The *cas9* cassette is cloned into a level-1 plasmid with connectors LS and R1 and the *csy4* cassette is cloned in a level 1 cassette with connectors L1 and RE. Both cassettes are cloned into the *HIS3* CEN/ARS entry vector with connectors LS' and RE' by BsaI Golden Gate assembly. **B.** The *cas9* cassette can be prepared according to the principles of a level-1 vector construction described by Lee et al[1]. It can encode either a regular Cas9 endonuclease or a dCas9/nCas9 variant fused to one (do 1) or two (do 1 + do 2) functional domains of choice, for applications such as transcription regulation or base editing. The part numbers in accordance with the MoClo toolkit are stated under each part.

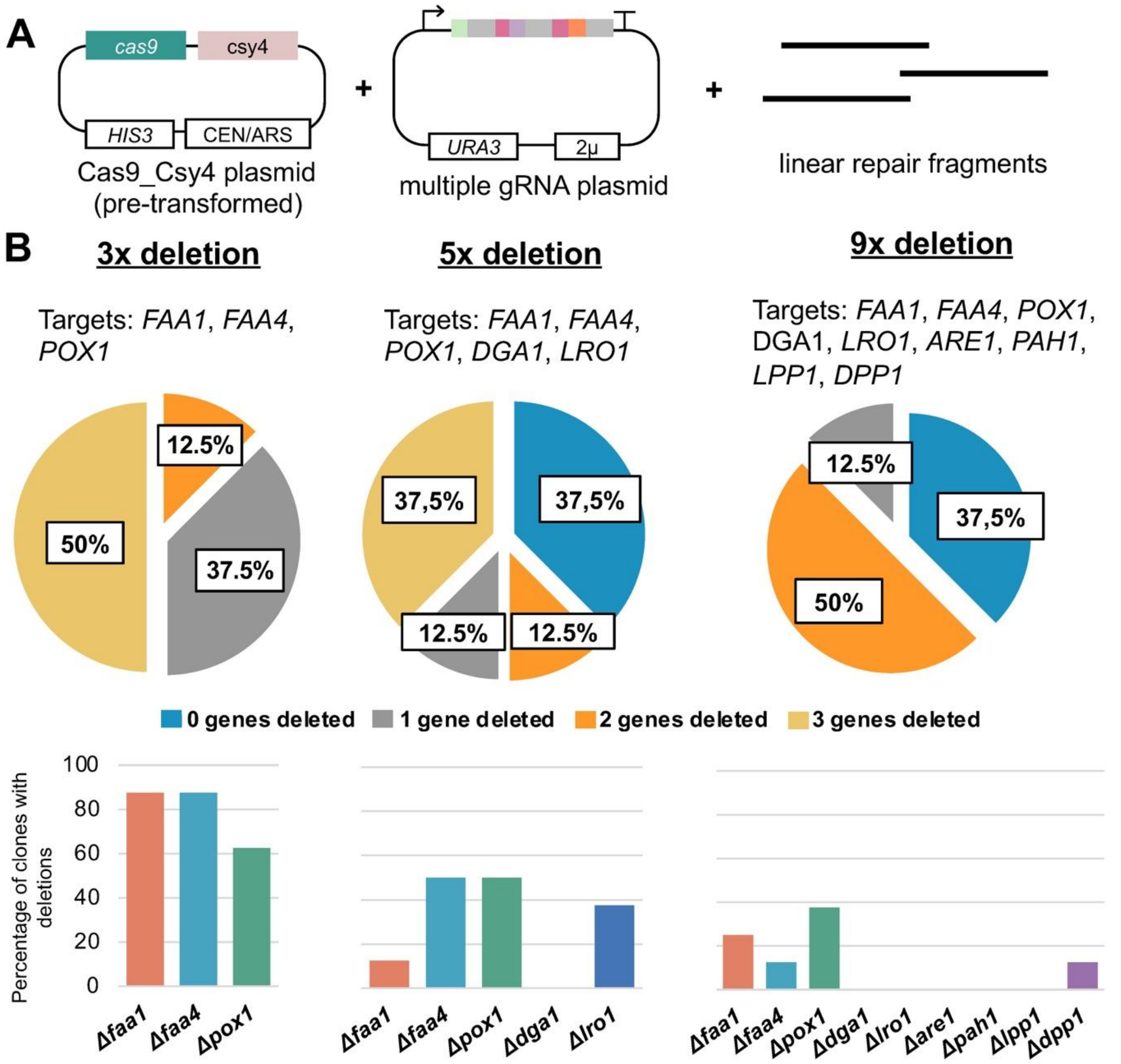

Supplementary Figure 2. Overview of the gene deletion approach using Csy4-multiplexed gRNA arrays. **A.** gRNA arrays targeting three (3x), five (5x) or nine (9x) genes were cloned into the vector pMCL9 with the order referred in the figure, and the resulting plasmids along with the corresponding DNA repair fragments were used to transform strains containing a Cas9_Csy4 plasmid. **B.** For each gene deletion assay, 8 yeast clones were screened for deletions. The pie charts represent the simultaneous deletions detected in each clone and in the bar charts represent the deletion frequency for each gene in each assay.

Supplementary Table 1. Plasmids constructed in this study

Name	Level/type	Description	*E.coli* marker	Yeast marker
pMC-X2	level-2	preassembled integration vector for X2 with type-234r GFP dropout, LS' RE'	Kan	-
pMC-X3	level-2	preassembled integration vector for X3 with type-234r GFP dropout, LS' RE'	Kan	-
pMC-X4	level-2	preassembled integration vector for X4 with type-234r GFP dropout, LS' RE'	Kan	-
pMC-XI1	level-2	preassembled integration vector for XI1 with type-234r GFP dropout, LS' RE'	Kan	-
pMC-XI2	level-2	preassembled integration vector for XI2 with type-234r GFP dropout, LS' RE'	Kan	-
pMC-XI3	level-2	preassembled integration vector for XI3 with type-234r GFP dropout, LS' RE'	Kan	-
pMC-XI5	level-2	preassembled integration vector for XI5 with type-234r GFP dropout, LS' RE'	Kan	-
pMC-XII1	level-2	preassembled integration vector for XII1 with type-234r GFP dropout, LS' RE'	Kan	-
pMC-XII2	level-2	preassembled integration vector for XII2 with type-234r GFP dropout, LS' RE'	Kan	-
pMC-XII4	level-2	preassembled integration vector for XII4 with type-234r GFP dropout, LS' RE'	Kan	-
pMC-XII5	level-2	preassembled integration vector for XII5 with type-234r GFP dropout, LS' RE'	Kan	-
pMC8b-X2up	level-0 / type-8b	containing 5' homologous region of X2	Cam	-
pMC8b-X3up	level-0 / type-8b	containing 5' homologous region of X3	Cam	-
pMC8b-X4up	level-0 / type-8b	containing 5' homologous region of X4	Cam	-
pMC8b-XI1up	level-0 / type-8b	containing 5' homologous region of XI1	Cam	-
pMC8b-XI2up	level-0 / type-8b	containing 5' homologous region of XI2	Cam	-
pMC8b-XI3up	level-0 / type-8b	containing 5' homologous region of XI3	Cam	-
pMC8b-XI5up	level-0 / type-8b	containing 5' homologous region of XI5	Cam	-
pMC8b-XII1up	level-0 / type-8b	containing 5' homologous region of XII1	Cam	-
pMC8b-XII2up	level-0 / type-8b	containing 5' homologous region of XII2	Cam	-
pMC8b-XII4up	level-0 / type-8b	containing 5' homologous region of XII4, medium-copy ori plasmid	Cam	-
pMC8b-XII5up	level-0 / type-8b	containing 5' homologous region of XII5	Cam	-
pMC7-X2dw	level-0 / type-7	containing 3' homologous region of X2	Cam	-
pMC7-X3dw	level-0 / type-7	containing 3' homologous region of X3	Cam	-
pMC7-X4dw	level-0 / type-7	containing 3' homologous region of X4	Cam	-

pMC7-XI1dw	level-0 / type-7	containing 3' homologous region of XI1	Cam	-
pMC7-XI2dw	level-0 / type-7	containing 3' homologous region of XI2	Cam	-
pMC7-XI3dw	level-0 / type-7	containing 3' homologous region of XI3	Cam	-
pMC7-XI5dw	level-0 / type-7	containing 3' homologous region of XI5	Cam	-
pMC7-XII1dw	level-0 / type-7	containing 3' homologous region of XII1	Cam	-
pMC7-XII2dw	level-0 / type-7	containing 3' homologous region of XII2	Cam	-
pMC7-XII4dw	level-0 / type-7	containing 3' homologous region of XII4	Cam	-
pMC7-XII5dw	level-0 / type-7	containing 3' homologous region of XII5	Cam	-
pMC6-spacer	level-0 / type-6	spacer containing non-coding DNA from pYTK048 instead of yeast marker	Cam	-
pMC234r-ccdB/TPK2	level-0 / type234r	dropout cassette containing toxic genes *ccdB* and *TEF1p-TPK2*, requires CcdB-resistant *E. coli* strain for amplification	Cam	-
pMC3-TPK2	leve-0 / type-3	containing the *TPK2* gene from *S. cerevisiae*	Cam	-
pMC234r-ccdB/TPK2-GAL	level-0 / type234r	dropout cassette containing toxic genes *ccdB* and *GAL1p-TPK2*, requires CcdB-resistant *E. coli* strain for amplification	Cam	-
pMC3-yeGFP	level-0 / type-3	containing *yeGFP*	Cam	-
pMC-Ura-Cen-lvl1	level-1	preassembled vector backbone with type-234r GFP dropout, LS R1 CEN/ARS	Amp	*URA3*
pMG332	level-1	*TEF1*p-BglII-*yeGFP-ADH1*t, LS R1 CEN/ARS	Amp	*URA3*
pMG333	level-1	*TEF1*p-*yeGFP-ADH1*t, LS R1 CEN/ARS	Amp	*URA3*
pMG343	level-1	preassembled vector backbone with toxic gene dropout (*ccdB* and *TEF1*p-*TPK2*), requires CcdB-resistant *E. coli* strain for amplification, CEN/ARS LS R1	Amp	*URA3*
pMG344	level-1	preassembled vector backbone with toxic gene dropout (*ccdB* and *GAL1*p-*TPK2*), requires CcdB-resistant *E. coli* strain for amplification, CEN/ARS LS R1	Amp	*URA3*
pMCL9	-	2µ cloning vector for single or multiplexed gRNAs	Amp	*URA3*
pMCL8	level-0 / type-234	gRNA cassette with *RPR1*p-tetO promoter and terminator *RPT1*t	Cam	-
pMCL8_28bp	level-0 / type-234	plasmid containing scaffold gRNA for Cas9 followed by a 28bp Csy4 recognition sequence	Cam	-
Csy4_2-4	level-0 / type-234	containing cassette *TEF1*p-Csy4-*CYC1*t	Cam	-
LS-Cas9-R1	level-1	*TEF1*p-Cas9-*ADE1*t LS R1 CEN/ARS	Kan	*HIS3*
L1-Csy4-RE	level-1	*TEF1*p-Csy4-*CYC1*t- t L1 RE CEN/ARS	Kan	*HIS3*
Cas9_Csy4	level-2	*TEF1*p-Cas9-*ADE1*t *TEF1*p-Csy4-*CYC1*t CEN/ARS	Amp	*HIS3*
3x_gRNA	-	pMCL9 with gRNAs for *FAA1, FAA4, POX1*	Amp	*URA3*
5x_gRNA	-	pMCL9 with gRNAs for *FAA1, FAA4, POX1, DGA1, LRO1*	Amp	*URA3*

9x_gRNA	-	pMCL9 with gRNAs for *FAA1, FAA4, POX1, DGA1, LRO1, ARE1, PAH1, LPP1, DPP1*	Amp	*URA3*
dCas9_3a	level-0 / type 3a	containing *dCas9* ORF with 4a ends	Cam	-

Supplementary Table 2. Summary of the MoClo integration plasmids constructed in this study with their corresponding single and triple gRNA plasmid, the genomic loci of each integration site and the verification primers for each site

MoClo integration plasmid	HRs amplified from	Correspon ding ECMF gRNA helper plasmid	Triple integration gRNA helper plasmid	Targeted genomic loci (sequence according to Mikkelsen *et al.*)	Verification primer pairs (and PCR product length)
pMC-X2	pCfB2899	pCfB3020		Chr X: 194944..195980	2220/902 (975 bp), 287/901 (674 bp)
pMC-X3	pCfB3034	pCfB3041	pCfB3051 (X3. XI2, XII2)	Chr X: 223616..224744	2220/904 (669 bp), 287/903 (858 bp)
pMC-X4	pCfB3035	pCfB3042	pCfB3052 (X4, XI3, XII5)	Chr X: 236336..237310	2220/906 (653 bp), 287/905 (784 bp)
pMC-XI1	pCfB3036	pCfB3043		Chr XI: 67491..68573	2220/908 (786 bp), 287/907 (591 bp)
pMC-XI2	pCfB2903	pCfB3044	pCfB3051 (X3. XI2, XII2)	Chr XI: 91575..92913	2220/910 (820 bp), 287/909 (764 bp)
pMC-XI3	pCfB2904	pCfB3045	pCfB3052 (X4, XI3, XII5)	Chr XI: 93378..94567	2220/912 (706 bp), 287/911 (728 bp)
pMC-XI5	pCfB3037	pCfB3046		Chr XI: 11779..118967	2220/8419 (703 kb), 287/8418 (851 bp)
pMC-XII1	pCfB3038	pCfB3047		Chr XII: 795787..796720	2220/892 (894 kb), 287/891 (653 kb)
pMC-XII2	pCfB3039	pCfB3048	pCfB3051 (X3. XI2, XII2)	Chr XII: 808805..809939	2220/894 (665 kb), 287/893 (596 kb)
pMC-XII5	pCfB2909	pCfB3050	pCfB3052 (X4, XI3, XII5)	Chr XII: 839226..840357	2220/900 (801 kb), 287/899 (612 kb)

All verification primers except for 287 from ECMF paper [2]

Supplementary Table 3. Comparison of ECMF integration vectors and MoClo integration vectors.

	average number of colonies	confirmed clones containing all 3 integrations
**triple integration MoClo plasmids**	19.5	3/5
**triple integration ECMF plasmids**	22.5	3/5

Two independent yeast transformations were performed using the gRNA helper vector pCfB3052 and the NotI-digested integration vectors pCfB2904, pCfB2909, pCfB3035 or their MoClo counter parts pMC-XI3, pMC-XII5, pMC-X4. Equimolar amounts of the “empty” integration vectors, containing bi-directonal terminators in case of the ECMF plasmids or GFP drop-out in case of the MoClo vectors, were transformed into CEN.PK113-11C carrying the *cas9*-containing plasmid pCfB2312. The table shows the average number of colonies on

YPD+G418+Nat plates after 3 d of incubation for two independent transformation experiments. 5 colonies of the MoClo and ECMF plates were analysed for correct integration using the primer pairs 287/899, 287/905 and 287/911 for the MoClo sample or the primer pairs 2221/899, 2221/905 and 2221/911 for the ECMF sample. Three out of 5 clones were found to have all 3 cassettes integrated, which is in the 60-70% targeting efficiencies range that was reported in the ECMF paper [2].

Supplementary Table 4. Primers used in the present study

Primer name	Sequence	Use
288	GCATCGTCTCATCGGTCTCACAATgcgctgagggtttaat	5' HR from ECMF vectors with MoClo overhangs
289	ATGCCGTCTCAGGTCTCAAGGGcgagctcgctgagga	
290	GCATCGTCTCATCGGTCTCAGAGTcctgcaggactagtgct	3' HR from ECMF vectors with MoClo overhangs
291	ATGCCGTCTCAGGTCTCATCGGcgctgaggtcttaat	
343	gatatcagtaatgagtcgaaaaagc	pCfB2988 remove BsmBI site 1
344	gctttttcgactcattactgatatc	
345	gcctgactttacgactccttcatgaat	pCfB2988 remove BsmBI site 2
346	attcatgaaggagtcgtaaagtcaggc	
347	cttatacccgactcttgacgctcgag	pCfB3034 remove BsmBI site
348	ctcgagcgtcaagagtcgggtataag	
349	aagatcaggacgactcgagcgctg	pCfB3036 remove BsmBI site
350	cagcgctcgagtcgtcctgatctt	
351	ttatgttctgagtcgtaagaaagggtg	pCfB3038 remove BsmBI site
352	caccctttcttacgactcagaacataa	
287	cgagctcgctgaggacttaa	universal cPCR reverse primer binding in MoClo integration vectors
374	GCATCGTCTCATCGGTCTCAGCTGACGCCGCGTGGATCCGGCTT	pMC5-ccdB
375	ATGCCGTCTCAGGTCTCATGTACAACGGAATGCGTGCGATcgc	
376	GTGTGCCGGTTTCCGTTATCGG	ccdB BsaI removal
377	CCGATAACGGAAACCGGCACAC	
368	GCATCGTCTCATCGGTCACAaacgtgagacccccacacaccatagcttcaaaatg	MC234r ccdB/TPK2 TEF1 fwd
372	GCATCGTCTCATCGGTCACAaacgtgagaccccgccccattatcttagccta	MC234r ccdB/TPK2 GAL1 fwd
378	ATGCCGTCTCAGGTCACAcagctgagacccCAACGGAATGCGTGCGATc	MC234r ccdB+TPK2 rev
TEF1p f OL BsaI	CCGAATggtctcaaacgccttgccaacagggagttc	p*TEF1* BglII site removal
TEF1p r -BglII OL BsaI	gcatagcaatctaatctaagttttaattacaaatatgtgagaccACGGGT	

MCL11	gcatcgtctcatcggtctcaaacgCAAAATGTTTCTACTCCTTTTTTACTCT	pTEF1-Csy4-CYC1t part 2-4
MCL12	atgccgtctcaggtctcacagcGCAAATTAAAGCCTTCGAGCGT	
MCL13	GCTGTGAGACCAGACCAATAAAAAAC	GFP marker from pYTK50 for pMCL8
MCL14	CGTTGGAGACCGACTACG	
MCL15	accgtagtcggtctccaacgGGGGATCTGCCAATTGAAC	pRPR1 for pMCL8
MCL16	ttccgtctcaCTGCCAATCGCAGCTCCC	
MCL19	tacgtctcagGTTTTAGAGCTAGAAATAGCAAG	sgRNA-RPR1t for pMCL8
MCL20	tattggtctggtctcacagcAAAGAACAACAAGAGTTCC	
MCL23	TCTGGATTTGTTCAGAACGCTCG	sequencing primer for pMCL8_28bp
MCL27	gcatggtctcaccctTCTTTGAAAAGATAATGTATGAT	pSNR52 for pMCL9
MCL28	atgcggtctcaGATCATTTATCTTTCACTGCGGA	
MCL30	gcatggtctcagatcTGAGACGGAAAGTGAAACGTG	GFP marker from pYTK50 for pMCL9
MCL32	atgcggtctcaaaacTGAGACGTATAAACGCAGAAAG	
MCL33	gcatggtctcaGTTTTAGAGCTAGAAATAGCAAG	SUP4t-CYC1t for pMCL9
MCL34	atgcggtctcatgtaGCAAATTAAAGCCTTCGAGCG	
Csy4_fw	gttcactgccgtataggcagctaagaaaCCATATCCAACTTCCAATTTAATCTTTCTT	add 28bp Csy4 recognition sequence to pMCL8
Csy4_rv	tttcttagctgcctatacggcagtgaacGCACCGACTCGGTGCCAC	
pmcl9_fw	gcattttttcacaccctacaatgtt	fw primer for colony PCR of pMCL9
pmcl9_rv_1	accaatctaagtctgtgctcc	rv primer for colony PCR of pMCL9
pmcl9_rv_2	gtgacataactaattacatgactcgaa	rv primer for colony PCR of pMCL9
FAA1_FAA4_fw	gcatcgtctcatttaaaagctaaaggtcttgatatcGTTTTAGAGCTAGAAATAGCAAGTTAA	fw primer with *FAA1* gRNA
FAA1_FAA4_rv	atgccgtctcaacttaaggtttattTTTCTTAGCTGCCTATACGGCAG	rv primer with first part of *FAA4* gRNA
FAA4_POX1_fw	gcatcgtctcaaagtttccacGTTTTAGAGCTAGAAATAGCAAGTTAA	fv primer with second part of *FAA4* gRNA
FAA4_POX1_rv	atgccgtctcactctaaagttgtTTTCTTAGCTGCCTATACGGCAG	rv primer with first part of *POX1* gRNA
POX1_DGA1_fw	gcatcgtctcaagagatctgatcGTTTTAGAGCTAGAAATAGCAAGTTAA	fv primer with second part of *POX1* gRNA
POX1_DGA1_rv	atgccgtctcattgttaatcatttTTTCTTAGCTGCCTATACGGCAG	rv primer with first part of *DGA1* gRNA
DGA1_LRO1_fw	gcatcgtctcaacaacatcatcGTTTTAGAGCTAGAAATAGCAAGTTAA	fv primer with second part of *DGA1* gRNA
DGA1_LRO1_rv	atgccgtctcatcagaatcTTTCTTAGCTGCCTATACGGCAG	rv primer with first part of *LRO1* gRNA

LRO1_ARE1_fw	gcatcgtctcactgatgaaaacaataaGTTTTAGAGCTAGAAATAGCAAGTTAA	fv primer with second part of *LRO1* gRNA
LRO1_ARE1_rv	atgccgtctcaaatcaaagtagtagTTTCTTAGCTGCCTATACGGCAG	rv primer with first part of *ARE1* gRNA
ARE1_PAH1_fw	gcatcgtctcagatttcaactGTTTTAGAGCTAGAAATAGCAAGTTAA	fv primer with second part of *ARE1* gRNA
ARE1_PAH1_rv	atgccgtctcacccccctcaatcaTTTCTTAGCTGCCTATACGGCAG	rv primer with first part of *PAH1* gRNA
PAH1_LPP1_fw	gcatcgtctcaggggcttgatgGTTTTAGAGCTAGAAATAGCAAGTTAA	fv primer with second part of *PAH1* gRNA
PAH1_LPP1_rv	atgccgtctcaggtaccagggatatctcTTTCTTAGCTGCCTATACGGCAG	rv primer with first part of *LPP1* gRNA
LPP1_DPP1_fw	gcatcgtctcatacctagGTTTTAGAGCTAGAAATAGCAAGTTAA	fv primer with second part of *LPP1* gRNA
LPP1_DPP1_rv	atgccgtctcagccaccatttttctccataaaggcTTTCTTAGCTGCCTATACGGCAG	rv primer with *DPP1* gRNA
POX1_rv	atgccgtctcagccagatcagatctctaaagttgtTTTCTTAGCTGCCTATACGGCAG	rv primer with *POX1* gRNA (3x plasmid)
LRO1_rv	atgccgtctcagccattattgttttcatcagaatcTTTCTTAGCTGCCTATACGGCAG	rv primer with *LRO1* gRNA (5x plasmid)
FAA1_dg fw	TACGAATGACACAGGGGCAC	Diagnostic primers for *FAA1* deletion
FAA1_dg rv	AAGGAGTCAGTGCACACCAG	
FAA4_dg fw	TGCAACCCCTCTGAGTTGAC	Diagnostic primers for *FAA4* deletion
FAA4_dg rv	TGCATAAATGAACGTGGGCG	
POX1_dg fw	GGAATTCGGTCATTAGCGGC	Diagnostic primers for *POX1* deletion
POX1_dg rv	ATCGTTGTGGCCGATGAAAC	
ARE1_dg fw	CCAACTCCTCAGGACGTGAC	Diagnostic primers for *ARE1* deletion
ARE1_dg rv	CTCTCGCGGAGGATACGTTC	
LRO1_dg fw	TCTGGCCCTTCAACTTCGAC	Diagnostic primers for *LRO1* deletion
LRO1_dg rv	GTACGTCGCTGCTGCAATTG	
DGA1_dg fw	GAAGTACTTCACCACGGGGG	Diagnostic primers for *DGA1* deletion
DGA1_dg rv	GCCTCTCAGTTACGCTTTGC	
PAH1_dg_F	GAAGAGTAGGCCATCGTGGG	Diagnostic primers for *PAH1* deletion
PAH1_dg_R	CAGCAAGCGAGTTTCGTGTG	
DPP1_dg_F	AGGGTCCACTAACATACGCG	Diagnostic primers for *DPP1* deletion
DPP1_dg_R	TCGCTTCAAGTTCGTTGCAC	
LPP1_dg_F	GCCACGTGAAACCTGACAAC	Diagnostic primers for *LPP1* deletion
LPP1_dg_R	CAGCTGTTTCACAGGCAACC	

Supplementary Table 5. Golden Gate assembly protocols used in this study (adapted from Engler *et al*. [3]). FD: Fast-Digest.

Step	Overnight protocol (>6 h) (FD enzymes recommended)		Quick protocol (2-3 h) (FD enzymes required)	
initial restriction (37°C)	4:00 min		4:00 min	
restriction (37°C)	2:00 min	50 cycles	1:00 min	30-50 cycles
ligation (16°C)	5:00 min		2:00 min	
final restriction (37°C) or final ligation (4°C) *	4:00 min			
heat inactivation (65°C)	10:00 min			
storage (12°C)	∞			

* Normally a final restriction step at 37°C is performed, except if a plasmid backbone containing a drop-out cassette is assembled (e.g. for the pMC integration backbones).

Supplementary Table 6. DNA repair oligos and gRNA spacers used in this study

Name	Sequence	Use
O1	GCATCGTCTCATCGGTCTCATACAactagagatctatgtgaggatcctaactcgagatcgaGAGTTGAGACCTGAGACGGCAT	MC 6 spacer fwd
O2	ATGCCGTCTCAGGTCTCAACTCtcgatctcgagttaggatcctcacatagatctctagtTGTATGAGACCGATGAGACGATGC	MC 6 spacer rev
FAA1_repair oligo fw	AAACTCGTTAGGATACAATAAAAACTAGAACAAACACAAAAGACAAAAAAAGACAACAATTGGATCAACATTTCCATGATAGGAAAGCCTCATCATACTAAAGCACTTTTTCAGTTTTTT	
FAA1_repair oligo rv	AAAAAACTGAAAAAGTGCTTTAGTATGATGAGGCTTTCCTATCATGGAAATGTTGATCCAATTGTTGTCTTTTTTTGTCTTTTGTGTTTGTTCTAGTTTTTATTGTATCCTAACGAGTTT	
FAA4_repair oligo fw	CACATCATTTTTTTCTCTGTTCTTCACTATTTCTTGAAAAACTAAGAAGTACGCATCAAAAGGAAGACATAGTTTTTTACTTTCCCCCCTGCCCTTCATAAACACTACGTTTCATTTTCT	
FAA4_repair oligo rv	AGAAAATGAAACGTAGTGTTTATGAAGGGCAGGGGGGAAAGTAAAAAACTATGTCTTCCTTTTGATGCGTACTTCTTAGTTTTTCAAGAAATAGTGAAGAACAGAGAAAAAAATGATGTG	
POX1_repair oligo fw	ACACTTGACACTAATAAGTATCACAGAAAAAAAGAAAATATAATAAATTAGTATTGCGATGTAGAGGTTTCCTGTTTTCCTTCGAACCCTCTGTTTTGCGACTTTTGTTTCAATTCAACT	
POX1_repair oligo rv	AGTTGAATTGAAACAAAAGTCGCAAAACAGAGGGTTCGAAGGAAAACAGGAAACCTCTACATCGCAATACTAATTTATTATATTTTCTTTTTTTCTGTGATACTTATTAGTGTCAAGTGT	
ARE1_repair oligo fw	ACTCACGCAGGTGGTTGTTCAGCACGGCTTGCAGCAAGAGCGCCAAAACAGATTGCAAGAACTGCCGCCATACCACGTGTGTCCCTCGCAAGCCCTTGATAGATATACAATAGGGAATGG	
ARE1_repair oligo rv	CCATTCCCTATTGTATATCTATCAAGGGCTTGCGAGGGACACACGTGGTATGGCGGCAGTTCTTGCAATCTGTTTTGGCGCTCTTGCTGCAAGCCGTGCTGAACAACCACCTGCGTGAGT	
LRO1_repair oligo fw	ATAGTAACAGCCATTACAAAAGGTTCTCTACCAACGAATTCGGCGACAATCGAGTAAAAAATGACCGACATTGACTCACTATCCATCCGTGTATTATTTCAAAGAGCGAAAAGAA	

	GGCGC	
LRO1_repair oligo rv	GCGCCTTCTTTTCGCTCTTTGAAATAATACACGGATGGATAGTGAGTCAATGTCGGTCATTTTTTACTCGATTGTCGCCGAATTCGTTGGTAGAGAACCTTTTGTAATGGCTGTTACTAT	
DGA1_repair oligo fw	CACATACACTTACATATACATAAGGAAACGCAGAGGCATACAGTTTGAACAGTCACATAATAATGAATTCATTGGAAAACACAAAATATGTTAGAATAAATAAGGATTTTTTAGTGTTTG	
DGA1_repair oligo rv	CAAACACTAAAAAATCCTTATTTATTCTAACATATTTTGTGTTTTCCAATGAATTCATTATTATGTGACTGTTCAAACTGTATGCCTCTGCGTTTCCTTATGTATATGTAAGTGTATGTG	
PAH1_repair_oligo_fw	TTTTACCTTCTAAGAAACATACAGGGAAGACATTACTGAAGATAGACACATCGGTCGATTAGATTCTTGTAGCCGAATATTATTTATAACGATCCATACTGCATATTAAGTAAAATTATG	
PAH1_repair_oligo_rv	CATAATTTTACTTAATATGCAGTATGGATCGTTATAAATAATATTCGGCTACAAGAATCTAATCGACCGATGTGTCTATCTTCAGTAATGTCTTCCCTGTATGTTTCTTAGAAGGTAAAA	
DPP1_repair_oligo_fw	GGGCACGTTATCAATTGTTAAAGGCAAAGAATCAGAATTAAATCATAGCAAACGACCAAAGAATAAAAAAGAATATATACTCCACATGACATACGAAATATACGTATTTATTGTTCTGTA	
DPP1_repair_oligo_rv	TACAGAACAATAAATACGTATATTTCGTATGTCATGTGGAGTATATATTCTTTTTTATTCTTTGGTCGTTTGCTATGATTTAATTCTGATTCTTTGCCTTTAACAATTGATAACGTGCCC	
LPP1_repair_oligo_fw	TTTCAAGAGTGAGAAACTCCTACATCAACGCCTAAGGAAACTCGTCATATTCTACCAAGGTAACACTTACAGAGTCCTATCAGGAAAGAATAAAAGCCGATCAAGCTTCATTCTCAGGTA	
LPP1_repair_oligo_rv	TACCTGAGAATGAAGCTTGATCGGCTTTTATTCTTTCCTGATAGGACTCTGTAAGTGTTACCTTGGTAGAATATGACGAGTTTCCTTAGGCGTTGATGTAGGAGTTTCTCACTCTTGAAA	
gRNA *faa1*	AAGCTAAAGGTCTTGATATC	
gRNA *faa4*	AATAAACCTTAAGTTTCCAC	
gRNA *pox1*	ACAACTTTAGAGATCTGATC	
gRNA *are1*	CTACTACTTTGATTTCAACT	
gRNA *lro1*	GATTCTGATGAAAACAATAA	
gRNA *dga1*	AAATGATTAACAACATCATC	
gRNA *pah1*	TGATTGAGGGGGGCTTGATG	
gRNA *dpp1*	GCCTTTATGGAGAAAAATGG	
gRNA *lpp1*	GAGATATCCCTGGTACCTAG	

Supplementary Note 1: PCR amplified sequence containing the *ccdB* cassette from pXII2-ccdB using the primers 374/375. Capital letters represent primer binding sequences.

CAACGGAATGCGTGCGATcgcagcctactcgctattgtcctcaatgccgtattaaatcataaaaagaaataagaaaa
agaggtgcgagcctcttttttgtgtgacaaaataaaaacatctacctattcatatacgctagtgtcatagtcctgaaaatcatctgcat
caagaacaatttcacaactcttatacttttctcttacaagtcgttcggcttcatctggattttcagcctctatacttactaaacgtgataaa
gtttctgtaatttctactgtatcgacctgcagactggctgtgtataagggagcctgacatttatattccccagaacatcaggttaatggc
gtttttgatgtcattttcgcggtggctgagatcagccacttcttccccgataacggagaccggcacactggccatatcggtggtcatc
atgcgccagctttcatccccgatatgcaccaccgggtaaagttcacgggagactttatctgacagcagacgtgcactggccagg
gggatcaccatccgtcgcccgggcgtgtcaataatatcactctgtacatccacaaacagacgataacggctctctcttttataggtg
taaaccttaaactgcatttcaccagcccctgttctcgtcagcaaaagagccgttcatttcaataaaccgggcgacctcagccatcc
cttcctgattttccgctttccagcgttcggcacgcagacgacgggcttcattctgcatggttgtgcttaccagaccggagatattgaca
tcatatatgccttgagcaactgatagctgtcgctgtcaactgtcactgtaatacgctgcttcatagcatacctctttttgacatacttcgg
gtatacatatcagtatatattcttataccgcaaaaatcagcgcgcaaatacgcatactgttatctggcttttagtAAGCCGGATC
CACGCGGCGT

References

(1) Lee, M. E.; DeLoache, W. C.; Cervantes, B.; Dueber, J. E. A Highly Characterized Yeast Toolkit for Modular, Multipart Assembly. *ACS Synth. Biol.* **2015**, *4* (9), 975–986. https://doi.org/10.1021/sb500366v.

(2) Jessop-Fabre, M. M.; Jakočiūnas, T.; Stovicek, V.; Dai, Z.; Jensen, M. K.; Keasling, J. D.; Borodina, I. EasyClone-MarkerFree: A Vector Toolkit for Marker-Less Integration of Genes into Saccharomyces Cerevisiae via CRISPR-Cas9. *Biotechnol. J.* **2016**, *11* (8), 1110–1117. https://doi.org/10.1002/biot.201600147.

(3) Engler, C.; Kandzia, R.; Marillonnet, S. A One Pot, One Step, Precision Cloning Method with High Throughput Capability. *PLOS ONE* **2008**, *3* (11). https://doi.org/10.1371/journal.pone.0003647.
